# Supplementary material for: Mutation of CMTR2 in Lung Adenocarcinoma Alters RNA Alternative Splicing and Reveals Therapeutic Vulnerabilities
Source: Nat Commun. 2025 Nov 6;16:9754. doi: 10.1038/s41467-025-64821-0 (PMC12592727; doi:10.1038/s41467-025-64821-0)
Supplement: Supplementary file 1 — Supplementary Information [file 41467_2025_64821_MOESM1_ESM.pdf]

Supplementary Information

Mutation of *CMTR2* in Lung Adenocarcinoma Alters RNA Alternative Splicing and Reveals Therapeutic Vulnerabilities

Table of contents

Supplementary Figure 1–21

Supplementary Table 1–8

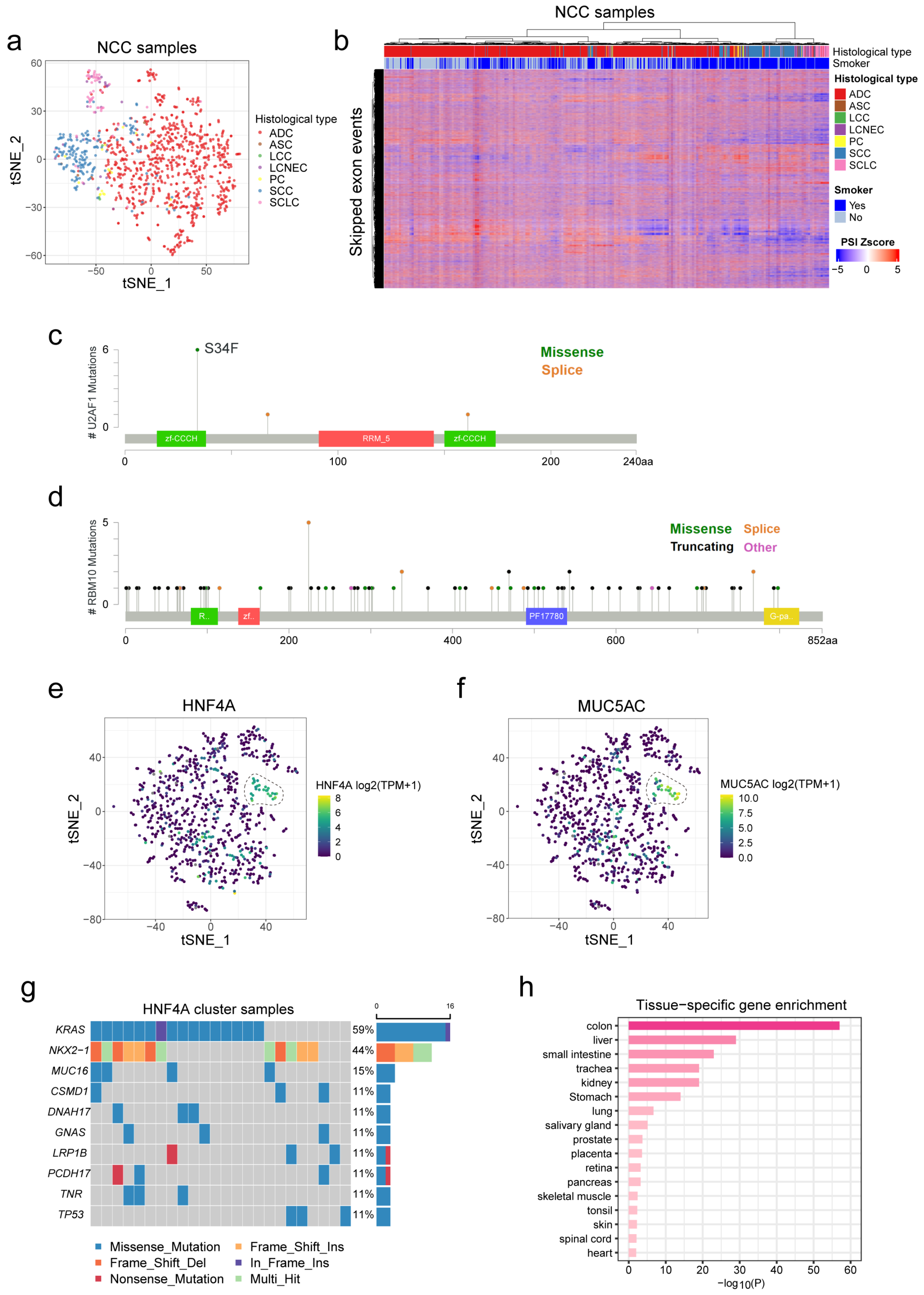

a
NCC samples
tSNE_1
tSNE_2
Histological type
ADC
ASC
LCC
LCNEC
PC
SCC
SCLC
b
NCC samples
Skipped exon events
Histological type
Smoker
Yes
No
PSI Zscore
c
U2AF1 Mutations
S34F
Missense
Splice
zf-CCCH
RRM_5
240aa
d
RBM10 Mutations
Missense
Splice
Truncating
Other
PF17780
852aa
e
HNF4A
HNF4A log2(TPM+1)
f
MUC5AC
MUC5AC log2(TPM+1)
g
HNF4A cluster samples
KRAS
NKX2−1
MUC16
CSMD1
DNAH17
GNAS
LRP1B
PCDH17
TNR
TP53
59%
44%
15%
11%
Missense_Mutation
Frame_Shift_Del
Nonsense_Mutation
Frame_Shift_Ins
In_Frame_Ins
Multi_Hit
h
Tissue−specific gene enrichment
colon
liver
small intestine
trachea
kidney
Stomach
lung
salivary gland
prostate
placenta
retina
pancreas
skeletal muscle
tonsil
skin
spinal cord
heart
−log10(P)

Supplementary Figure 1. U2AF1, RBM10, and HNF4A cluster within the AS landscape of LADC.

a Landscape of AS for all histological types in the NCC lung cancer cohort samples analyzed based on SE PSI values. Each t-SNE plot reflects the reduction of each sample in the higher-dimensional splice event PSI matrix to two dimensions. **b** Hierarchical clustering and heat-map analysis of differential SE events in samples of all histological types from the NCC lung cancer cohort. Rows and columns represent SE events and samples, respectively. Z-scores in the matrix correspond to normalized PSI values. **c**, **d** Lollipop plots depicting mutations in *U2AF1* (c) and *RBM10* (d) in the NCC LADC cohort. **e, f** t-SNE plots of LADC samples from the NCC cohort, analyzed based on SE PSI values and color-coded according to the expression level of *HNF4A* (e) and *MUC5AC* (f). **g** Oncoplot showing the top 10 most frequently mutated genes in the HNF4A cluster. The bar chart on the right shows the frequency of each mutation type for each gene in this cluster. **h** Tissue/cell-specific gene signature enrichment analysis of upregulated genes in the HNF4A cluster. The horizontal bar graph shows enriched signatures based on PaGenBase data.

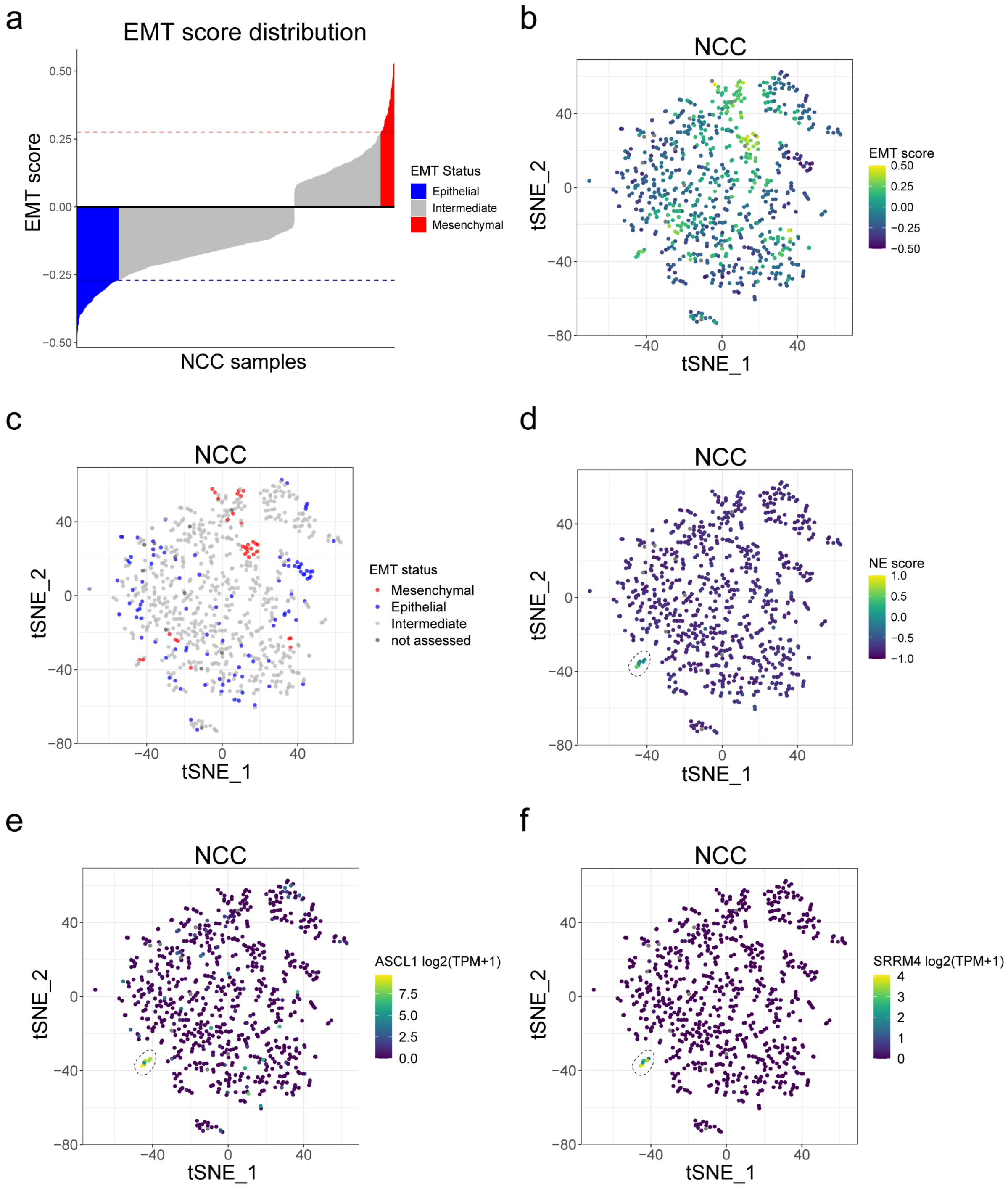

Supplementary Figure 2. EMT and neuroendocrine clusters in the AS landscape of LADC.

a Distribution of EMT scores calculated using the Kolmogorov-Smirnov test with the established EMT gene signature across NCC samples. EMT status was classified as epithelial (score < 0, FDR < 0.05), mesenchymal (score > 0, FDR < 0.05), or intermediate (all other samples). **b**–**f** t-SNE plots of LADC samples in the NCC

cohort analyzed according to SE PSI values and color-coded by EMT score (b), EMT status (c), neuroendocrine (NE) score calculated by single-sample GSEA using the established NE gene signature (d), and the expression levels of *ASCL1* (e) and *SRRM4* (f).

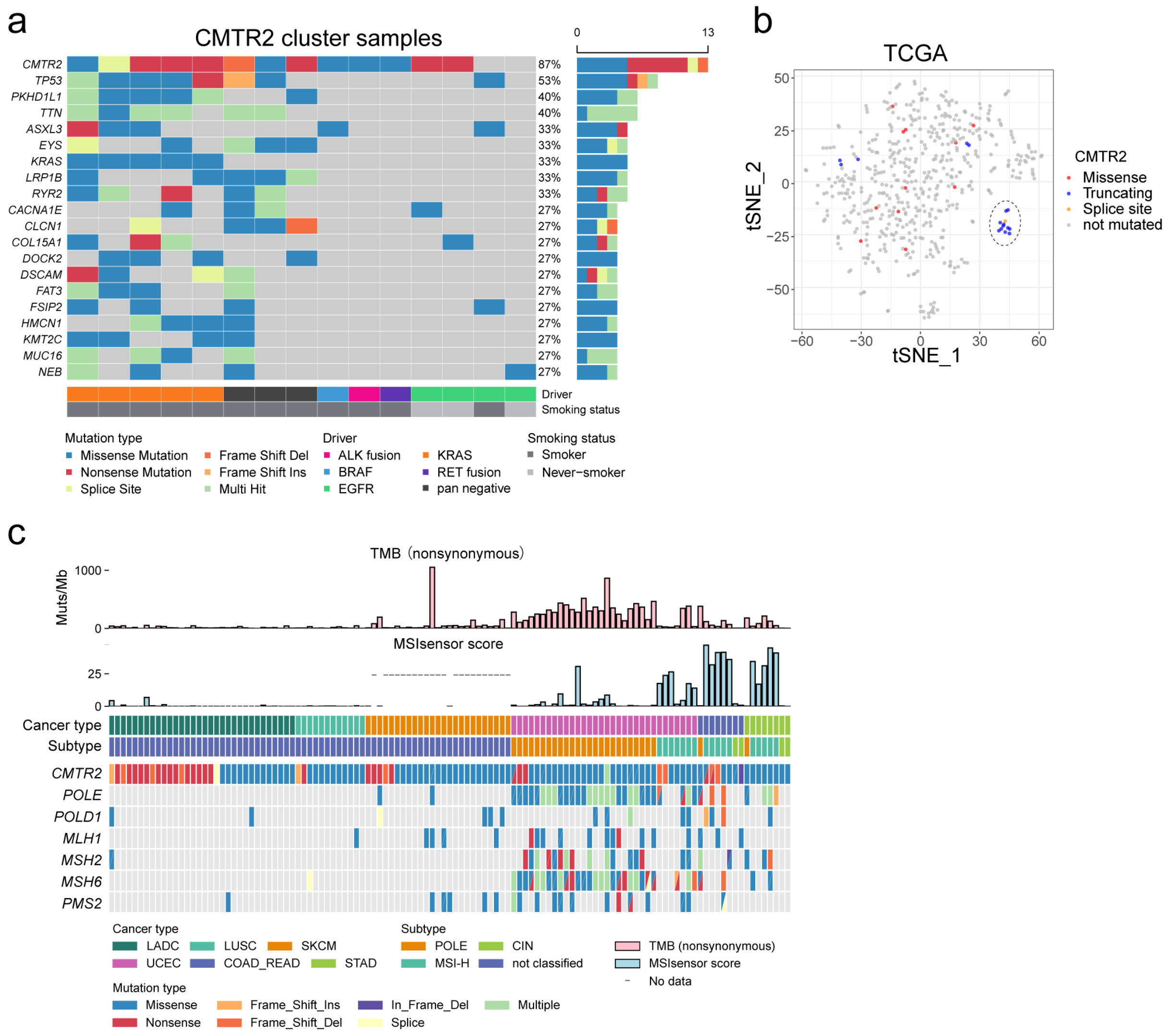

Supplementary Figure 3. Genomic characteristics of *CMTR2*-mutated samples in LADC and other cancers.

a Oncoplot showing the top 20 most frequently mutated genes in the CMTR2 cluster. The bar chart on the right shows the frequency of each mutation type in each gene in this cluster. **b** t-SNE plots of LADC samples from the TCGA cohort analyzed based on SE PSI values, which are color-coded according to *CMTR2* mutation type. **c** Oncoplot of *CMTR2*-mutated samples obtained from TCGA cancer types with a high *CMTR2* mutation frequency. Columns show *CMTR2* mutations and mutations associated with hypermutated subtypes (i.e., *POLE*, *POLD1*, and DNA mismatch repair genes). Abbreviations: CIN, chromosomal instability; COAD_READ, colon adenocarcinoma and rectum adenocarcinoma; LADC, lung adenocarcinoma; LUSC, lung squamous carcinoma;

MSI-H, microsatellite instability-high; SKCM, skin cutaneous melanoma; STAD, stomach adenocarcinoma; TMB, tumor mutation burden; UCEC, uterine corpus endometrial carcinoma.

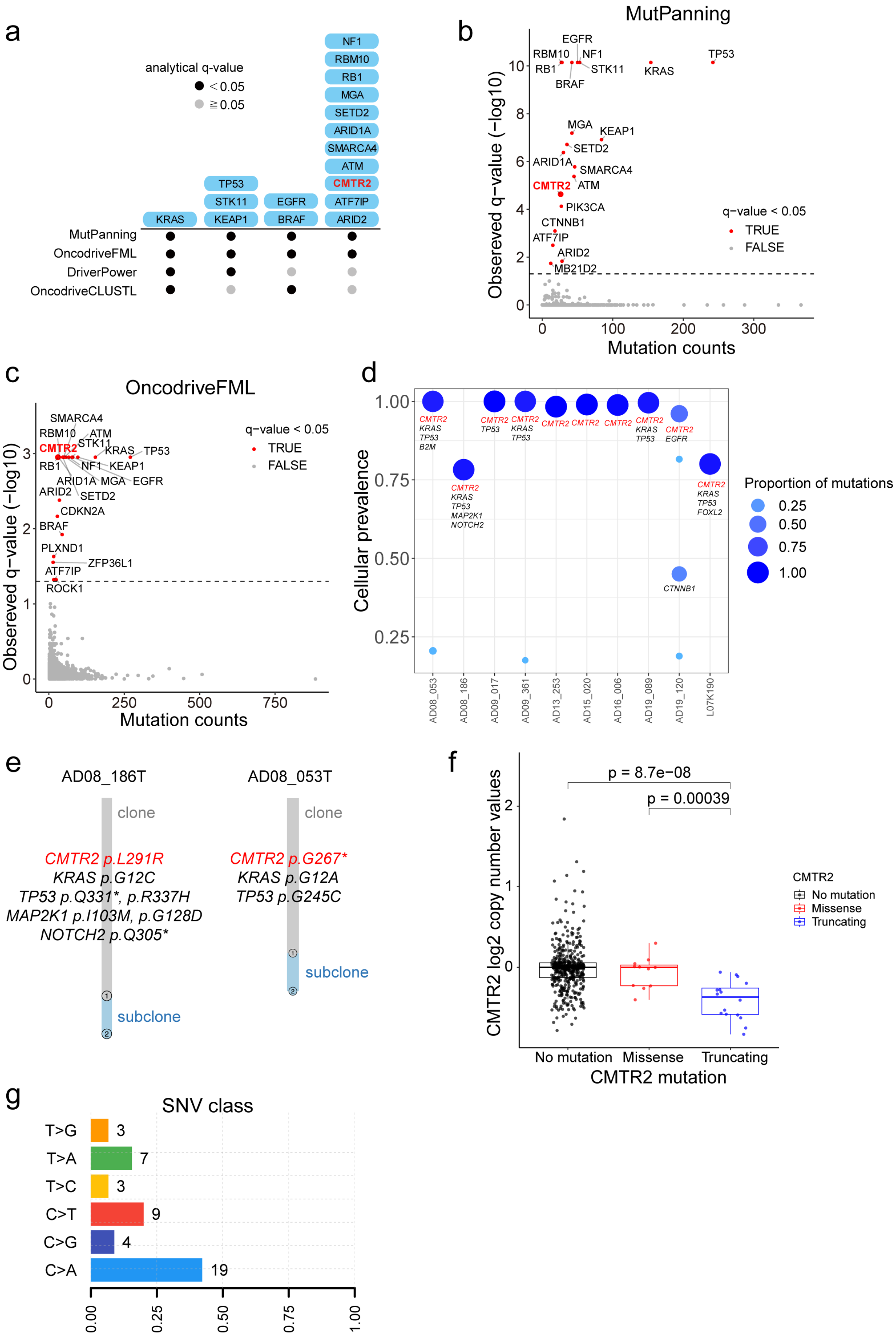
a
analytical q-value
＜0.05
≧0.05
NF1
RBM10
RB1
MGA
SETD2
ARID1A
SMARCA4
ATM
CMTR2
ATF7IP
ARID2
TP53
STK11
KEAP1
EGFR
BRAF
KRAS
MutPanning
OncodriveFML
DriverPower
OncodriveCLUSTL
b
MutPanning
Obsereved q−value (−log10)
Mutation counts
q−value < 0.05
TRUE
FALSE
EGFR
RBM10
NF1
RB1
STK11
BRAF
KRAS
TP53
MGA
KEAP1
SETD2
ARID1A
SMARCA4
CMTR2
ATM
PIK3CA
CTNNB1
ATF7IP
ARID2
MB21D2
c
OncodriveFML
Obsereved q−value (−log10)
Mutation counts
q−value < 0.05
TRUE
FALSE
SMARCA4
RBM10
ATM
STK11
CMTR2
KRAS
TP53
RB1
NF1
KEAP1
ARID1A
MGA
EGFR
ARID2
SETD2
CDKN2A
BRAF
PLXND1
ZFP36L1
ATF7IP
ROCK1
d
Cellular prevalence
Proportion of mutations
0.25
0.50
0.75
1.00
CMTR2
KRAS
TP53
B2M
MAP2K1
NOTCH2
EGFR
FOXL2
CTNNB1
AD08_053
AD08_186
AD09_017
AD09_361
AD13_253
AD15_020
AD16_006
AD19_089
AD19_120
L07K190
e
AD08_186T
clone
CMTR2 p.L291R
KRAS p.G12C
TP53 p.Q331*, p.R337H
MAP2K1 p.I103M, p.G128D
NOTCH2 p.Q305*
subclone
AD08_053T
clone
CMTR2 p.G267*
KRAS p.G12A
TP53 p.G245C
subclone
f
p = 8.7e−08
p = 0.00039
CMTR2 log2 copy number values
CMTR2
No mutation
Missense
Truncating
CMTR2 mutation
g
SNV class
T>G 3
T>A 7
T>C 3
C>T 9
C>G 4
C>A 19
0.00
0.25
0.50
0.75
1.00

Supplementary Figure 4. Identification of *CMTR2* as a putative driver gene in LADC.

a Putative driver genes in the TCGA LADC cohort identified using four computational tools: MutPanning, OncodriveFML, DriverPower, and OncodriveCLUSTL. For each gene, black circles indicate it was identified as a statistically significant driver (q-value < 0.05) by the corresponding tool, while light gray circles indicate non-significance (q-value ≥ 0.05). **b**, **c** Putative driver genes in the TCGA LADC cohort identified using MutPanning (b) and OncodriveFML (c). The x- and y-axes represent the mutation counts and the statistical significance [$-\log_{10}$(q-value)] of each gene, respectively. **d** Mutation clusters (clones) in *CMTR2*-mutated cases (SNVs only) in the CMTR2 cluster. The size of each dot corresponds to the proportion of somatic mutations in the respective clone. **e** Estimated clone structures in two cases in the CMTR2 cluster from the NCC cohort. **f** Box plot showing *CMTR2* mutation types and their corresponding copy number values in the TCGA LADC cohort. Statistical significance was assessed using the two-sided Wilcoxon rank-sum test. **g** SNV classes in the *CMTR2* gene in the NCC and TCGA LADC cohorts. x-axis: relative fraction of each substitution type. y-axis: observed substitution patterns.

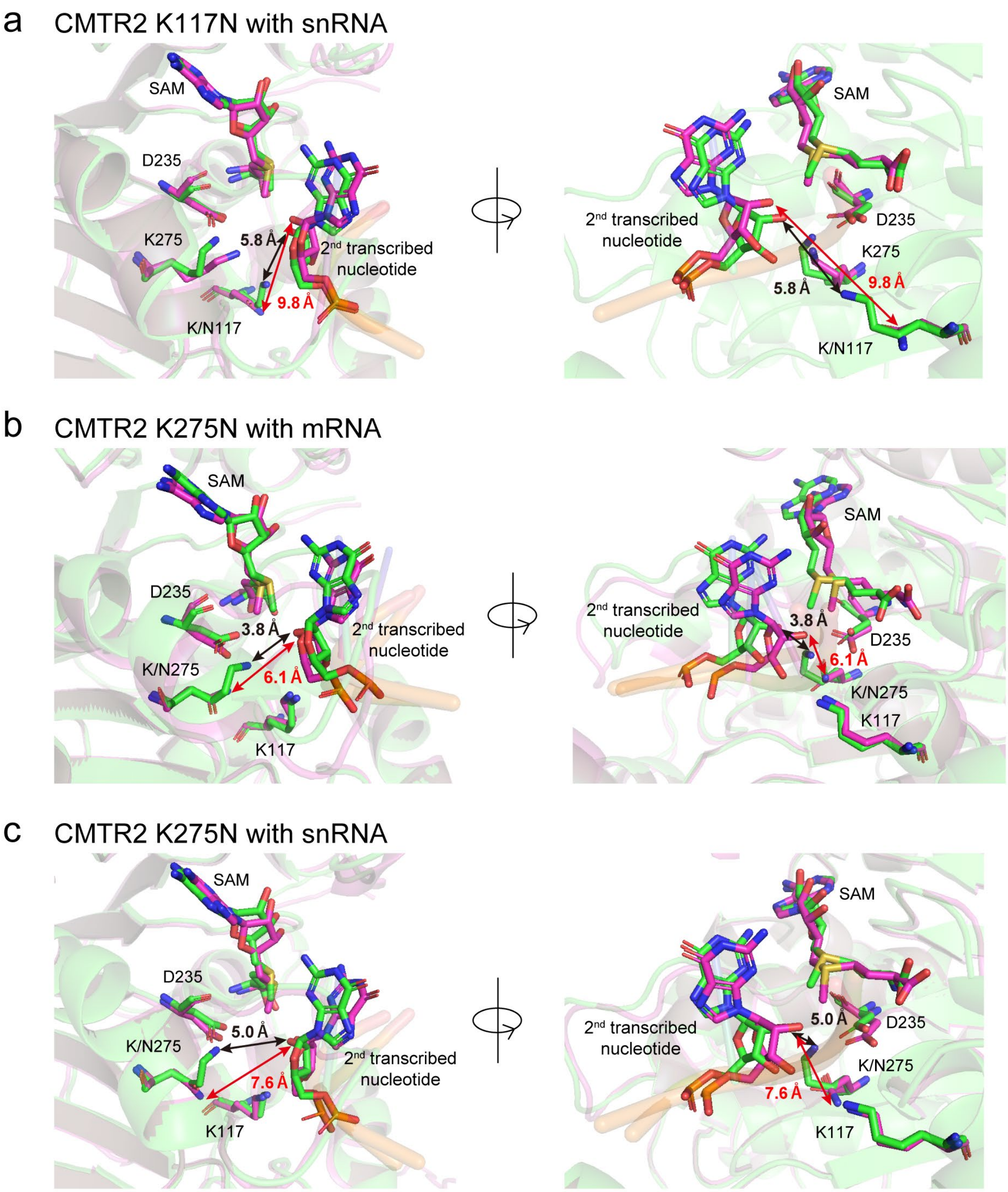

Supplementary Figure 5. MD structures of (a) the K117N mutant complexed with snRNA/SAM, (b) the K275N mutant complexed with mRNA/SAM, and (c) the K275N mutant complexed with snRNA/SAM. The mean structure obtained from five × 1-μs MD simulations of each mutant (magenta) was superimposed on that of WT CMTR2 (green). The main chains of the protein and RNA are shown as a transparent ribbon model, while the catalytic K-D-K motif residues (K/N117, D235, and K/N275), SAM, and the second transcribed nucleotide of mRNA/snRNA substrates are highlighted with stick models. Black and red arrows indicate the

distances between the amino nitrogen of K/N117 (K/N275) and the 2'-O position of the second transcribed nucleotide, respectively.

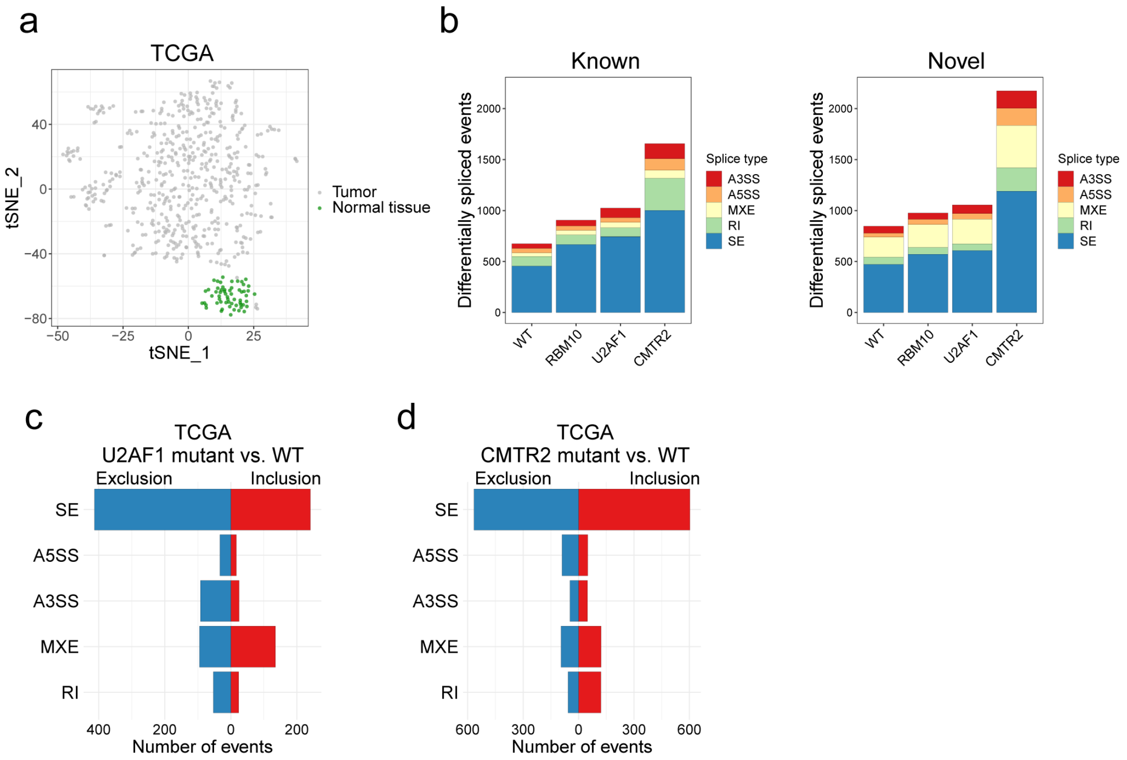

Supplementary Figure 6. RNA splicing alterations caused by *CMTR2* deficiency in the TCGA cohort.

a t-SNE plots of LADC (tumor) samples and normal tissues from the TCGA cohort analyzed according to SE PSI values. **b** Stacked bar charts showing the count and type of differentially spliced events identified by comparing normal tissues with WT tumors and *RBM10*-, *U2AF1*-, and *CMTR2*-mutated samples; $n = 10$ each. Samples were obtained at random from each cluster in the TCGA cohort. Spliced events were defined as those with $|\Delta PSI| \geq 0.1$ and FDR <0.05. Left: Known (annotated) spliced events. Right: Novel (unannotated) spliced events based on known or novel splice sites. **c**, **d** Bilateral bar charts depicting the number of significant differentially spliced events between *U2AF1* (c) or *CMTR2* (d) mutant and WT tumors in the TCGA cohort ($n = 10$ per group). *CMTR2* mutant samples were selected from those in the CMTR2 cluster that did not have a combination of other splicing factor mutations (FDR < 0.05; red: inclusion events ($\Delta PSI \geq 0.1$); blue: exclusion events ($\Delta PSI \leq -0.1$)).

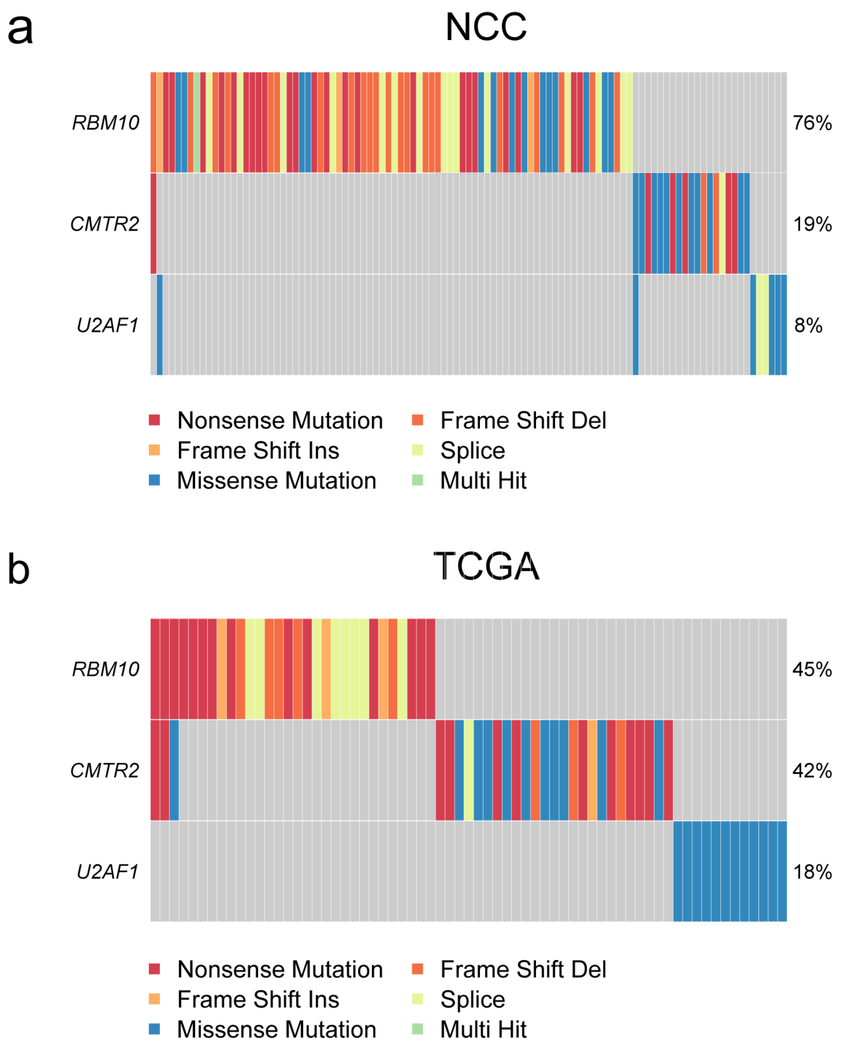

Supplementary Figure 7. Oncoplots showing *RBM10*-, *CMTR2*-, and *U2AF1*-mutated samples in the NCC (a) and TCGA (b) LADC cohorts.

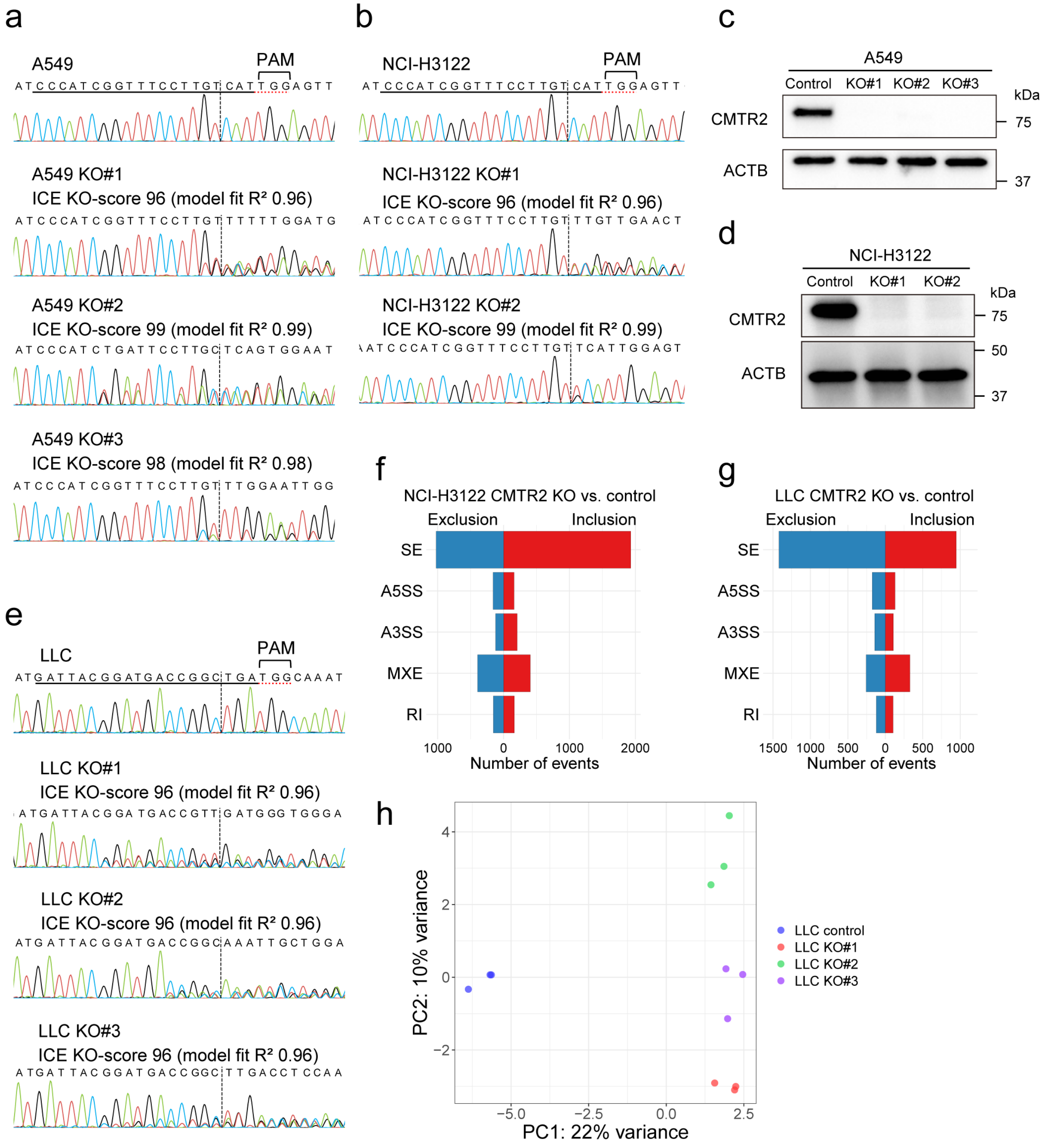

Supplementary Figure 8. Validation of *CMTR2* knockout and splicing alterations.

a, b, e Sanger sequencing chromatograms, with ICE knockout (KO) scores confirming CRISPR-mediated knockout of *CMTR2* in A549 (a), NCI-H3122 (b), and LLC (e) cells. Analysis was performed using the ICE analysis tool. **c, d** Western blot confirming knockout of *CMTR2* in A549 (c) and NCI-H3122 (d) cells (representative of two independent experiments). **f, g** Bilateral bar charts depicting the number of significant

differentially spliced events between *CMTR2*-knockout and control NCI-H3122 (f) and LLC (g) cells (FDR < 0.05; red: inclusion events [ΔPSI ≥ 0.1); blue: exclusion events (ΔPSI ≤ -0.1)]. **h** PCA plot indicating differences in AS between *CMTR2*-knockout clones and control cells. Each dot represents one of the three replicates obtained for each clone or control cells. PCA analysis was performed using the PSI value of each cell.

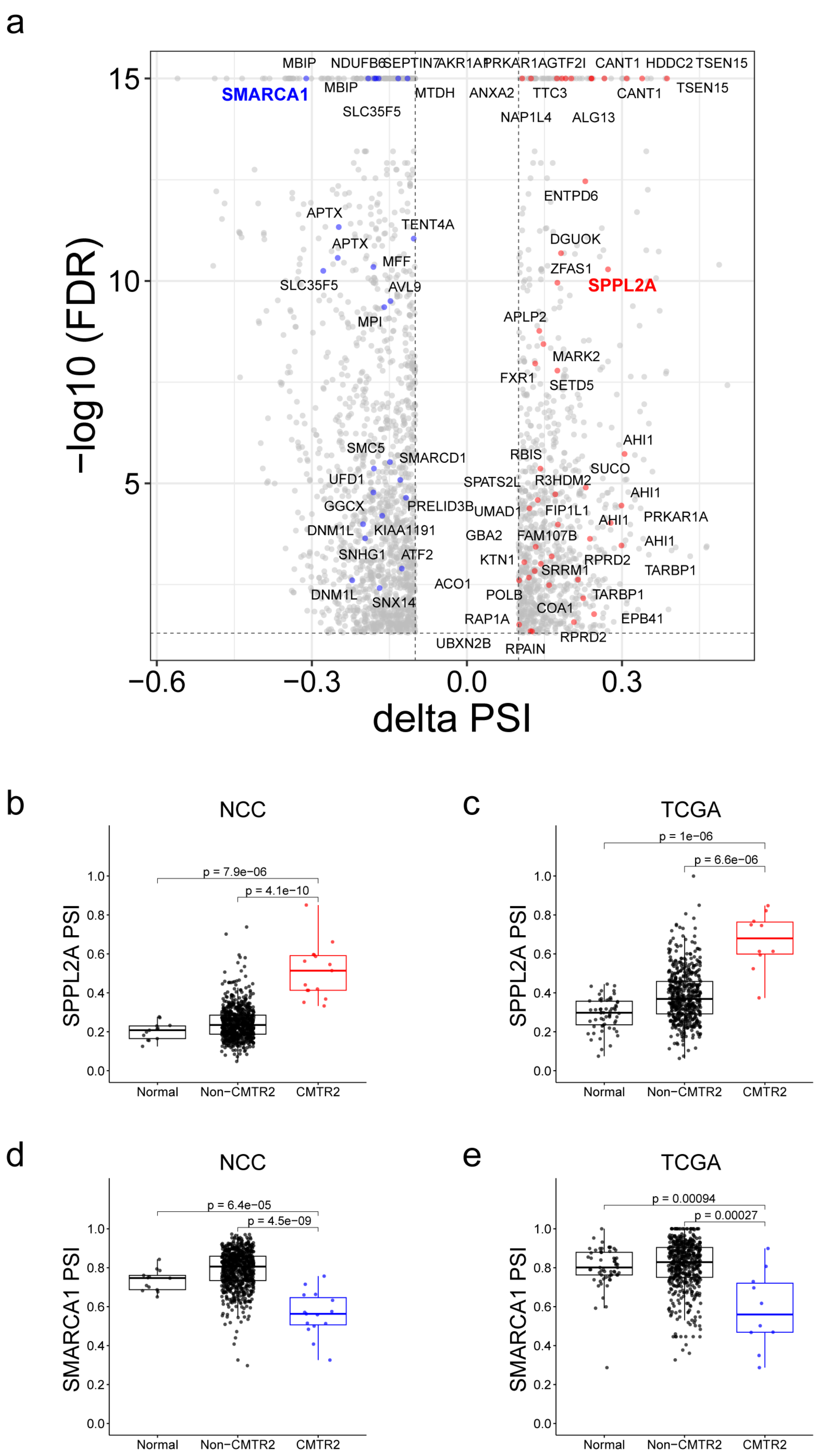
a
SMARCA1
SPPL2A
MBIP
NDUFB6
SEPTIN7
AKR1A1
PRKAR1A
GTF2I
CANT1
HDDC2
TSEN15
MTDH
ANXA2
TTC3
NAP1L4
ALG13
SLC35F5
ENTPD6
DGUOK
ZFAS1
APTX
TENT4A
MFF
AVL9
MPI
APLP2
MARK2
FXR1
SETD5
SMC5
SMARCD1
RBIS
AHI1
SUCO
UFD1
SPATS2L
R3HDM2
GGCX
PRELID3B
UMAD1
FIP1L1
PRKAR1A
DNM1L
KIAA1191
GBA2
FAM107B
SNHG1
ATF2
KTN1
SRRM1
RPRD2
TARBP1
ACO1
POLB
COA1
SNX14
RAP1A
EPB41
UBXN2B
RPAIN
−log10 (FDR)
delta PSI
b
NCC
SPPL2A PSI
p = 7.9e−06
p = 4.1e−10
c
TCGA
p = 1e−06
p = 6.6e−06
d
NCC
SMARCA1 PSI
p = 6.4e−05
p = 4.5e−09
e
TCGA
p = 0.00094
p = 0.00027
Normal
Non−CMTR2
CMTR2

Supplementary Figure 9. Consistent *CMTR2* mutation-induced SE events in human tumor samples and knockout cell lines.

a Volcano plot showing differential SE events in *CMTR2* mutant *versus* WT samples in the NCC cohort. Colored dots indicate the 71 common SE events identified in both human tumor samples and *CMTR2*-knockout cell lines. **b–e** Boxplots depicting the PSI values for *SPPL2A* (b, c) and *SMARCA1* (d, e) in normal tissues, non-CMTR2 tumors (outside the CMTR2 cluster), and CMTR2 tumors (within the CMTR2 cluster) from the NCC (b, d) and TCGA (c, e) cohorts. The center line indicates the median value, the lower and upper hinges represent the 25th and 75th percentiles, respectively, and the whiskers denote the 1.5× interquartile range. Statistical analysis was performed using the two-sided Wilcoxon rank-sum test.

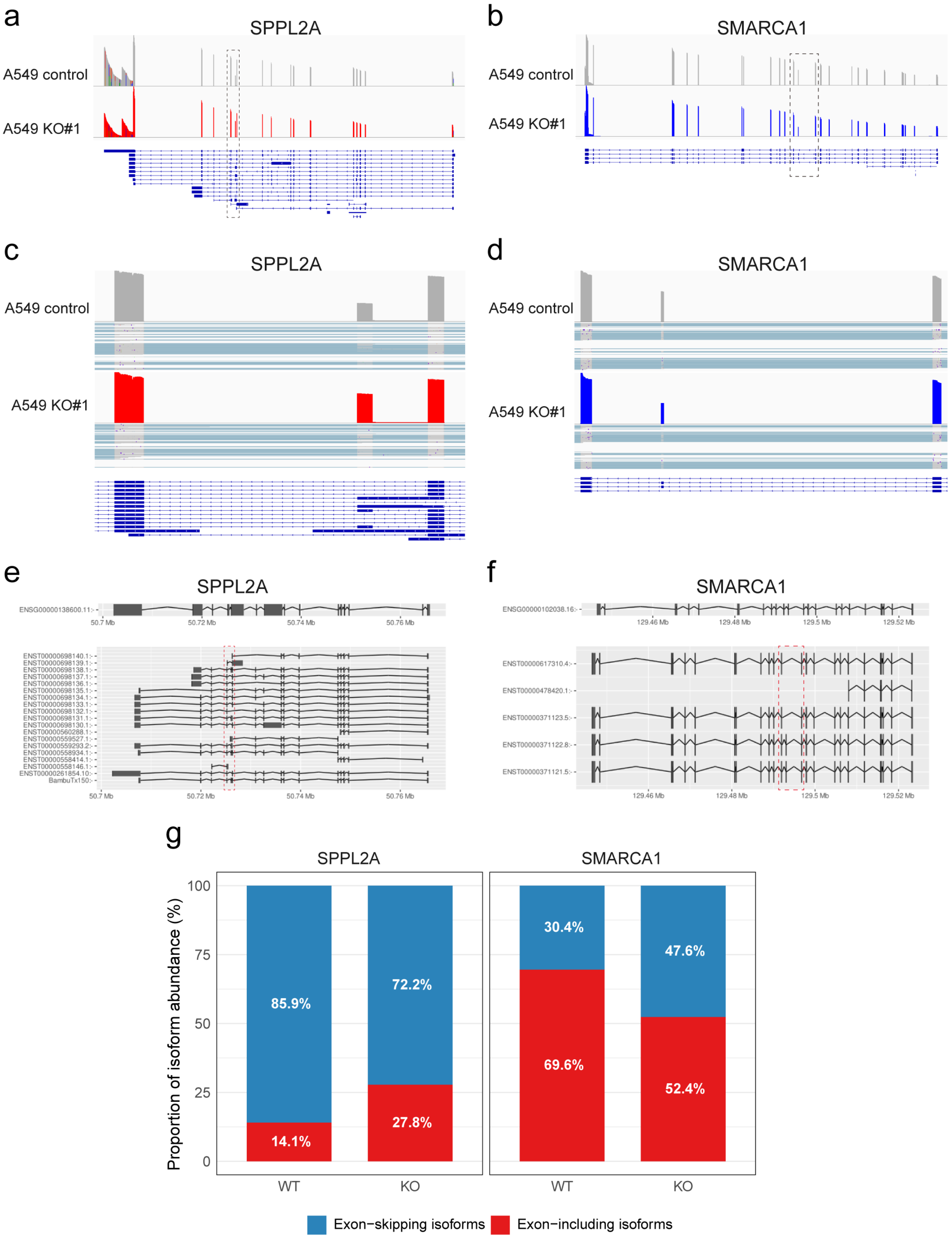
a
SPPL2A
A549 control
A549 KO#1
b
SMARCA1
A549 control
A549 KO#1
c
SPPL2A
A549 control
A549 KO#1
d
SMARCA1
A549 control
A549 KO#1
e
SPPL2A
f
SMARCA1
g
SPPL2A
SMARCA1
Proportion of isoform abundance (%)
100
75
50
25
0
85.9%
14.1%
72.2%
27.8%
30.4%
69.6%
47.6%
52.4%
WT
KO
WT
KO
Exon−skipping isoforms
Exon−including isoforms

Supplementary Figure 10. Long-read direct RNA-seq validation of splicing events in *CMTR2*-knockout cells.

a–d Long-read direct RNA-seq coverage plots of A549 *CMTR2*-knockout and control cells. Full-length transcript visualization of *SPPL2A* (a) and *SMARCA1* (b). Magnified views of differentially spliced junctions in *SPPL2A* (c) and *SMARCA1* (d). **e–f** Annotated transcript isoform structures for *SPPL2A* (e) and *SMARCA1* (f), with differentially spliced regions highlighted in red. BambuTx150 of *SPPL2A* represents a novel isoform detected by Bambu. **g** Relative abundances of transcript isoforms containing or lacking the highlighted exons for *SPPL2A* and *SMARCA1* in A549 *CMTR2*-knockout (KO) and WT cells.

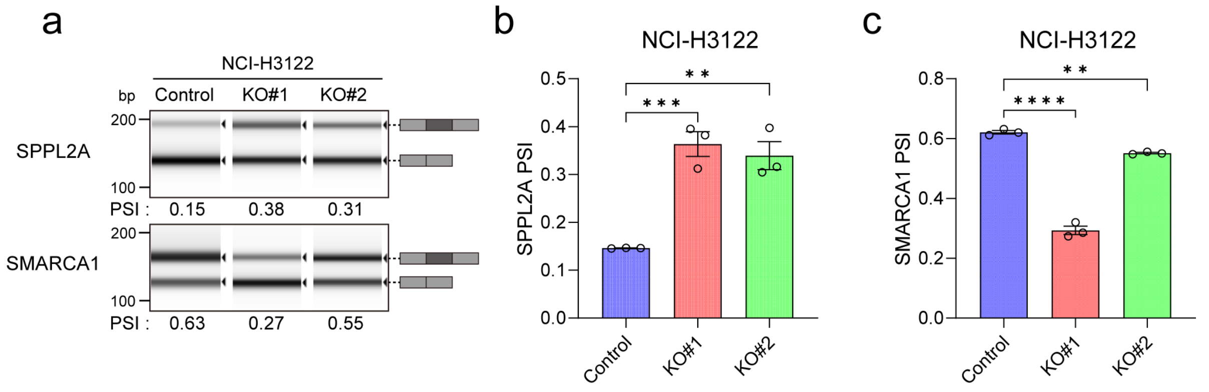

Supplementary Figure 11. RT-PCR validation of differentially spliced events in *SPPL2A* and *SMARCA1* upon *CMTR2* knockout in NCI-H3122 cells.

a Agilent TapeStation gel-like images of RT-PCR products. **b, c** Quantification of PSI values for *SPPL2A* (b) and *SMARCA1* (c). $n = 3$ independent experiments per condition. Error bars, ± SEM. Statistical significance was determined by a one-way ANOVA followed by Dunnett's multiple comparison test. *SPPL2A*: $^{**}p = 0.0016$, $^{***}p = 0.0009$; *SMARCA1*: $^{**}p = 0.0024$, $^{****}p < 0.0001$.

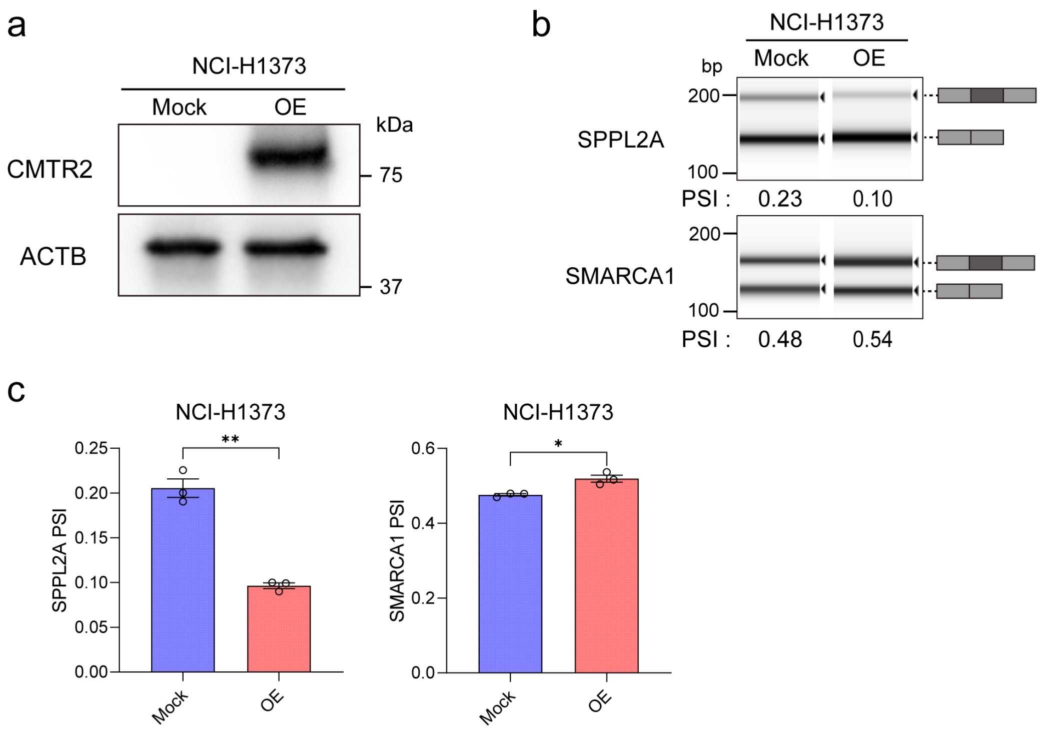

Supplementary Figure 12. Rescue experiment demonstrating reversal of the splicing changes in *SPPL2A* and *SMARCA1*.

a Western blot analysis confirming transduction of CMTR2 into NCI-H1373 cells using a lentiviral vector (NCI-H1373-OE) (representative of two independent experiments). **b** Agilent TapeStation gel-like images of RT-PCR products validating differentially spliced events in *SPPL2A* and *SMARCA1* in NCI-H1373-OE cells. Data are presented as a comparison with the mock control. **c** Quantification of PSI values for *SPPL2A* (left) and *SMARCA1* (right). $n = 3$ independent experiments per condition. Error bars, ± SEM. Statistical significance was determined by the two-sided Welch's t-test. *SPPL2A*: $^{**}p = 0.0054$; *SMARCA1*: $^{*}p = 0.0345$.

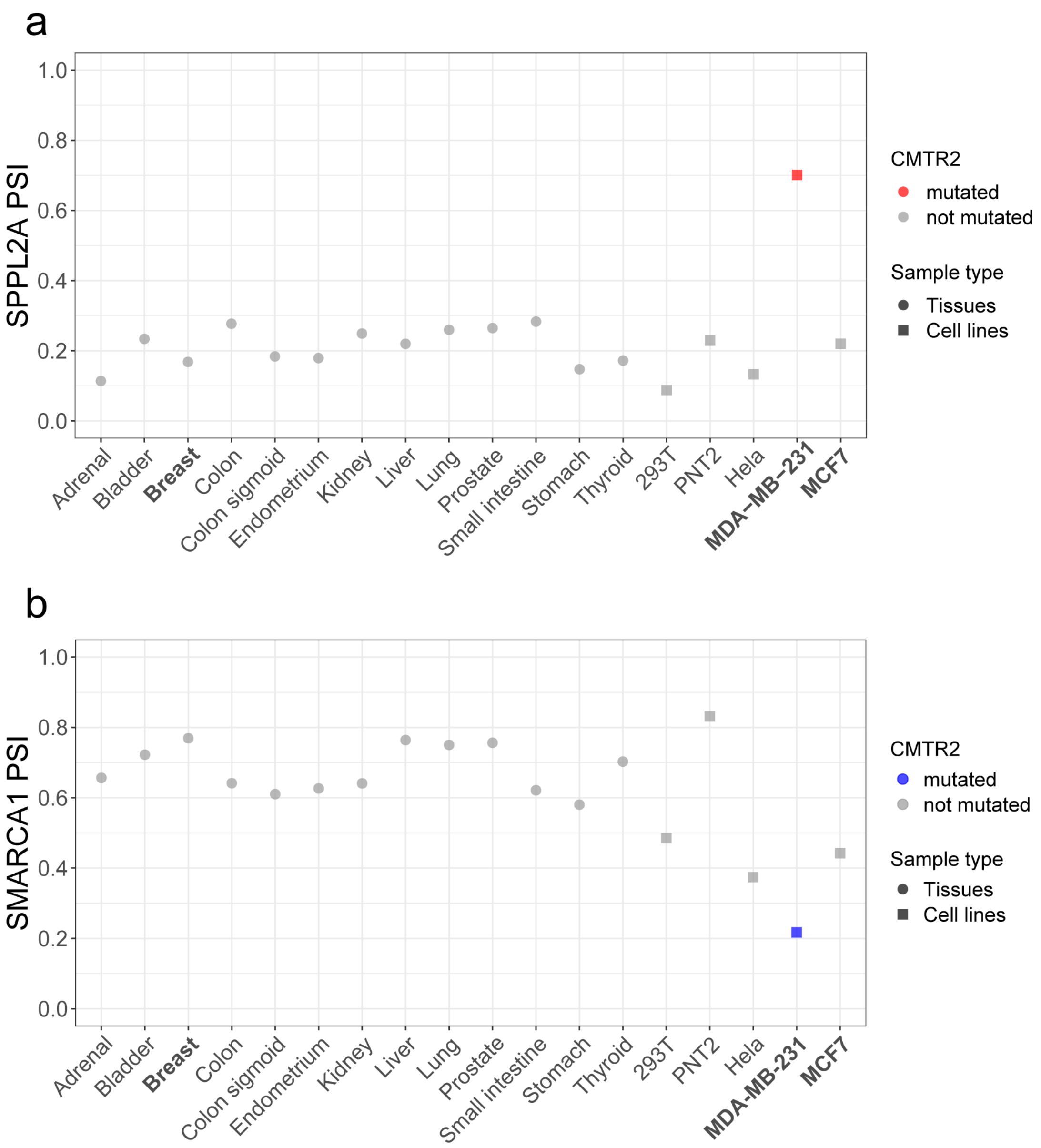

Supplementary Figure 13. PSI values for *SPPL2A* and *SMARCA1* SE events in normal tissues and cell lines.

a *SPPL2A*. **b** *SMARCA1*. Normal breast tissue and breast cancer cell lines are shown in bold.

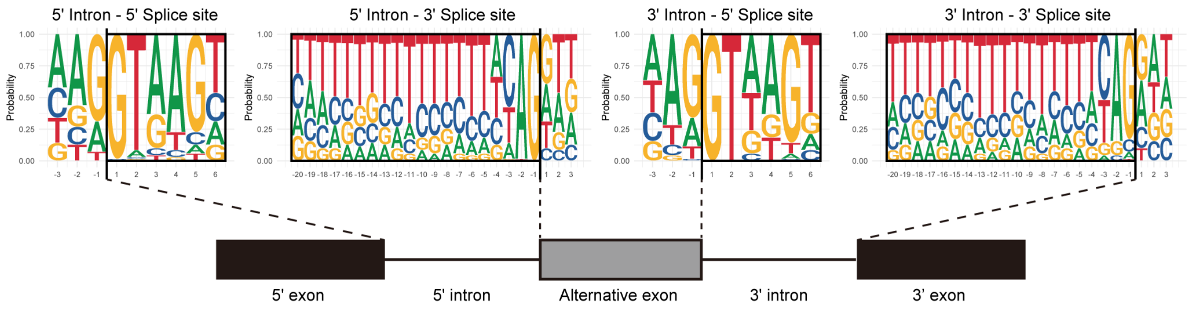

Supplementary Figure 14. Splice-site sequence motifs of the 71 shared SE events altered by *CMTR2* deficiency.

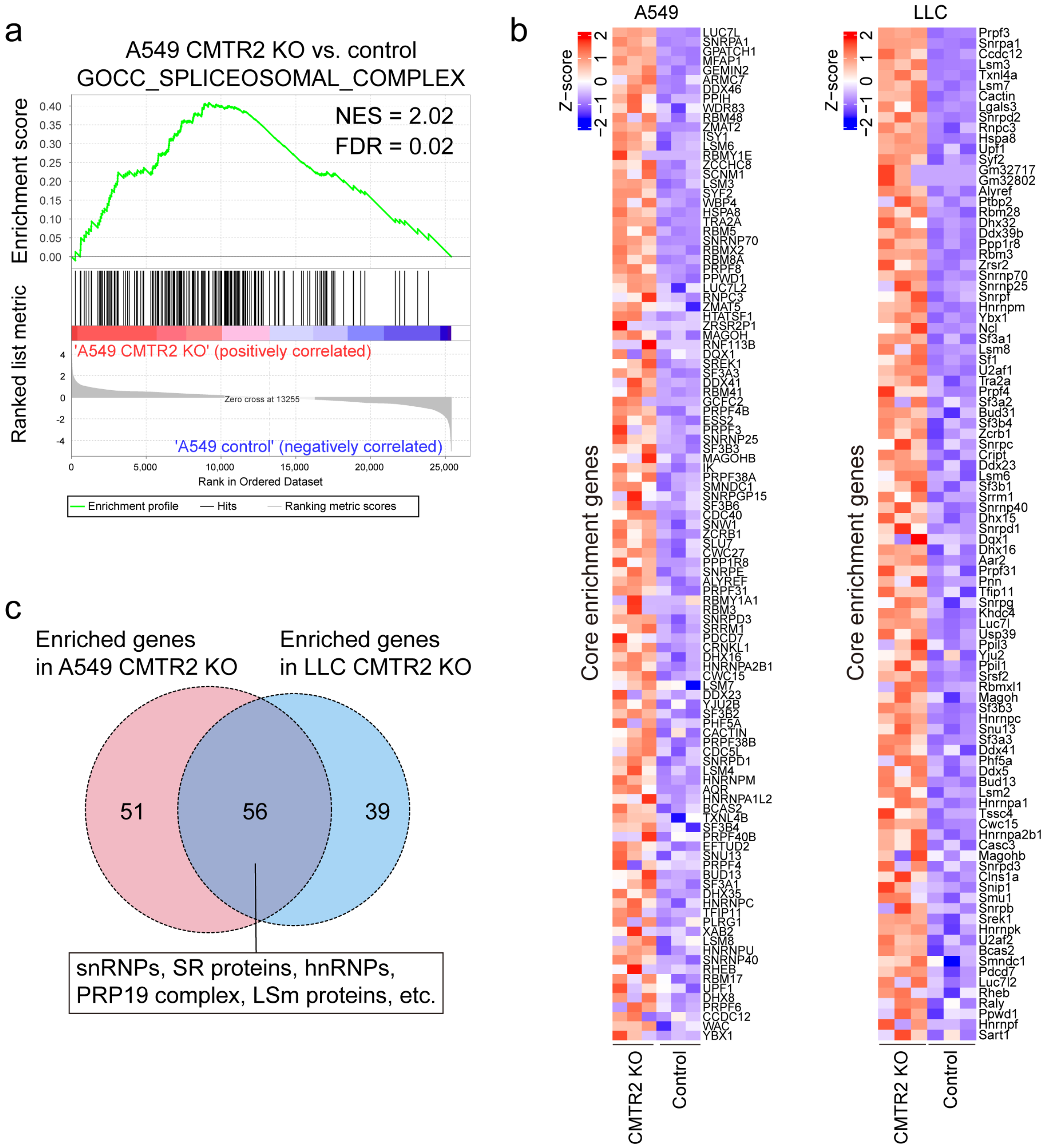

Supplementary Figure 15. Alterations in expression of spliceosomal complex components caused by *CMTR2* knockout.

a GSEA enrichment plots of the SPLICEOSOMAL COMPLEX gene set in A549 cells comparing *CMTR2* knockout and control conditions. **b** Heat-map showing the expression levels of core enrichment genes from the SPLICEOSOMAL COMPLEX gene set in A549 (left) and LLC (right) *CMTR2*-knockout clones and control cells. **c** Venn diagram showing overlap of core enrichment genes from the SPLICEOSOMAL COMPLEX gene

set enriched in A549 (human) and LLC (mouse) *CMTR2*-knockout clones. Orthologous genes were compared between the two species.

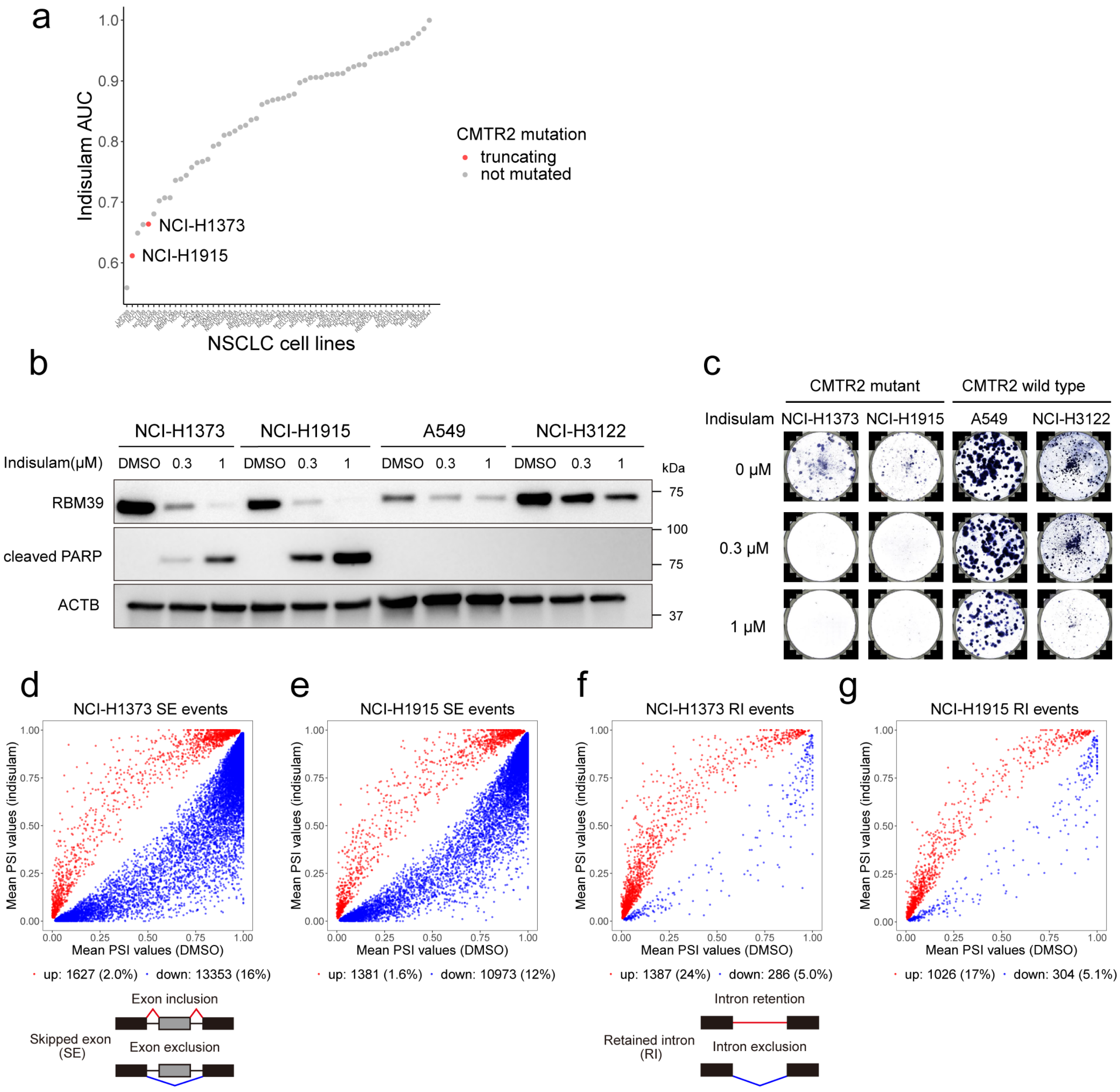

Supplementary Figure 16. Sulfonamide-induced growth suppression and splicing changes in *CMTR2*-deficient lung cancer cells.

a Sensitivity (AUC) to indisulam in NSCLC cell lines included in the PRISM Repurposing Secondary Screen. **b** Western blot analysis of CMTR2-expressing A549 and NCI-H3122 cells and *CMTR2*-deficient NCI-H1373 and NCI-H1915 cells treated for 72 h with increasing doses of indisulam (representative of two independent experiments). **c** Colony formation assay using CMTR2-expressing A549 and NCI-H3122 cells and *CMTR2*-

deficient NCI-H1373 and NCI-H1915 cells treated for 14 days with indisulam (representative of two independent experiments). **d–g** Scatter plots comparing the mean PSI values for NCI-H1373 (d, f) and NCI-H1915 (e, g) cells treated with 1 μM indisulam or DMSO for 24 h. Significantly associated events (FDR < 0.05) are color-coded in red or blue. SE events (d, e). Retained intron events (f, g).

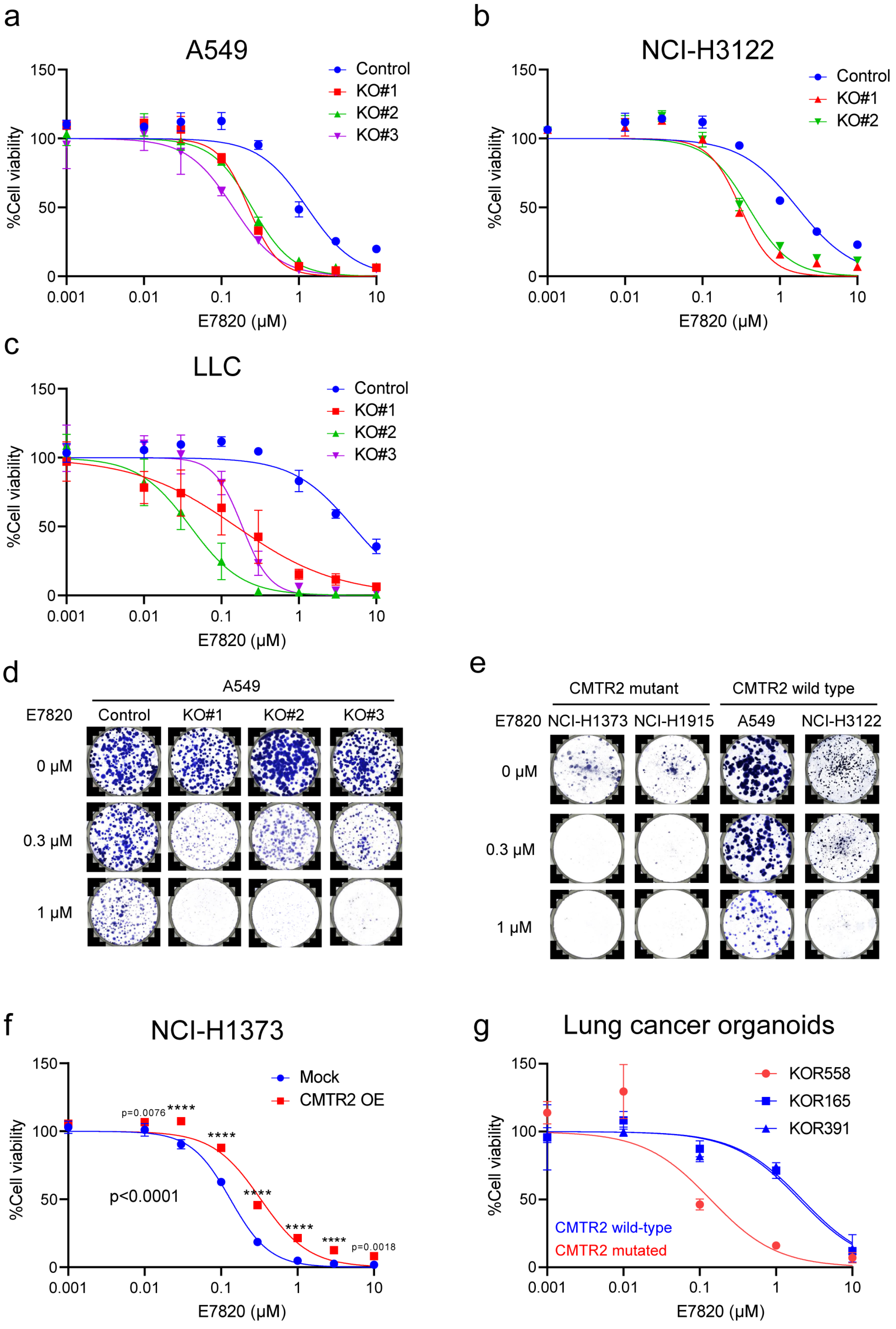
a
A549
%Cell viability
E7820 (μM)
Control
KO#1
KO#2
KO#3
b
NCI-H3122
%Cell viability
E7820 (μM)
Control
KO#1
KO#2
c
LLC
%Cell viability
E7820 (μM)
Control
KO#1
KO#2
KO#3
d
A549
E7820
Control
KO#1
KO#2
KO#3
0 μM
0.3 μM
1 μM
e
CMTR2 mutant
CMTR2 wild type
E7820
NCI-H1373
NCI-H1915
A549
NCI-H3122
0 μM
0.3 μM
1 μM
f
NCI-H1373
%Cell viability
E7820 (μM)
Mock
CMTR2 OE
p<0.0001
p=0.0076
p=0.0018
g
Lung cancer organoids
%Cell viability
E7820 (μM)
KOR558
KOR165
KOR391
CMTR2 wild-type
CMTR2 mutated

Supplementary Figure 17. Growth suppression of *CMTR2*-deficient cells by the splicing modulator E7820

a–**c** Drug-response curves of CRISPR-mediated *CMTR2*-knockout clones and control A549 (a), NCI-H3122 (b), and LLC (c) cell lines treated with E7820 for 5 days (*n* = 4 technical replicates; mean ± SD). **d** Colony formation assay using CRISPR-mediated *CMTR2*-knockout clones and control A549 cells treated with E7820 for 11 days (representative of two independent experiments). **e** Colony formation assay using CMTR2-expressing A549 and NCI-H3122 cells and *CMTR2*-deficient NCI-H1373 and NCI-H1915 cells treated with E7820 for 14 days (representative of two independent experiments). **f** Drug-response curves of NCI-H1373 cells transduced with CMTR2 (CMTR2-OE) or the empty vector control after treatment with E7820 for 5 days (*n* = 4 technical replicates; mean ± SD). The *P*-value was calculated by a two-way ANOVA. Asterisks indicate significant differences between the two cell lines at each concentration (Bonferroni's multiple comparisons test; ****$p <$ 0.0001). **g** Drug-response curves of lung cancer organoids harboring a *CMTR2* nonsense mutation (KOR558) and of *CMTR2* WT (KOR165 and KOR391) organoids treated with E7820 for 7 days (*n* = 4 technical replicates; mean ± SD).

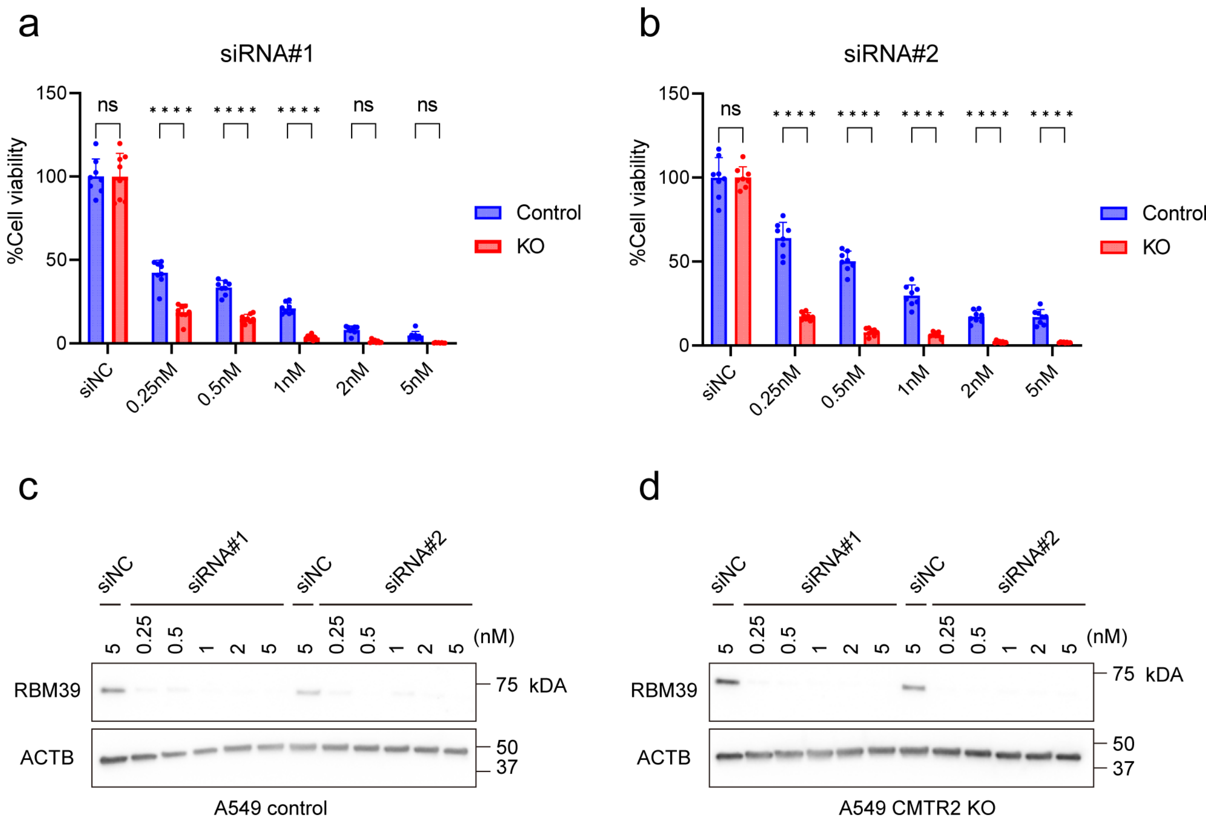

Supplementary Figure 18. Enhanced sensitivity of *CMTR2*-knockout cells to *RBM39*-targeting siRNA.

a, b Relative viability of CRISPR-mediated CMTR2-knockout clones and control A549 cells following treatment with *RBM39*-targeting siRNA#1 (a) and siRNA#2 (b) at the indicated concentrations. Data represent mean ± SD from n = 8 technical replicates. Statistical significance was determined by a two-way ANOVA followed by Bonferroni's multiple comparisons test. NS, not significant; ****$p < 0.0001$ between cell lines at each concentration. **c, d** Western blot analysis of RBM39 protein expression levels in A549 control (c) and *CMTR2*-knockout (d) cells following siRNA treatment at the indicated concentrations (representative of two independent experiments).

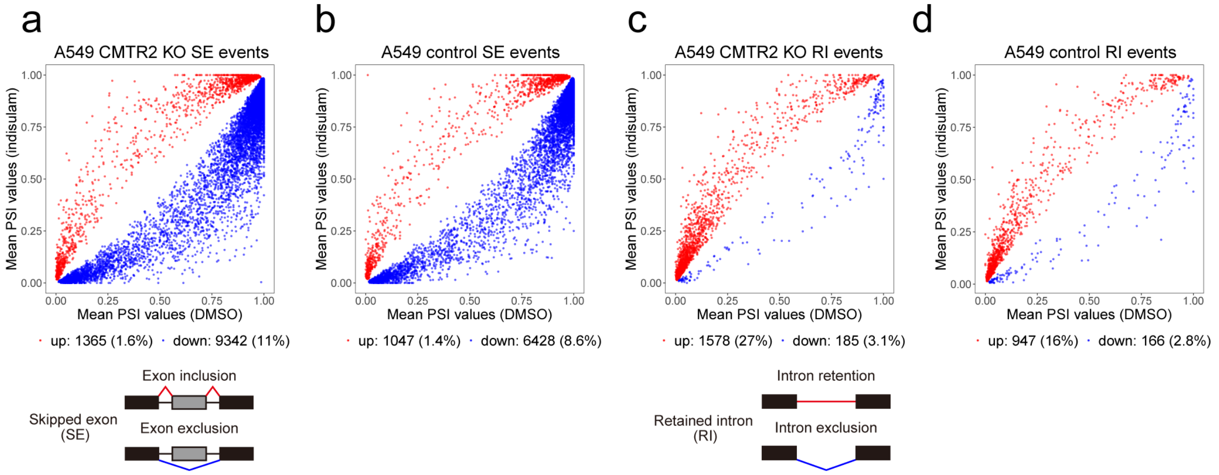

Supplementary Figure 19. Sulfonamide-induced splicing changes in *CMTR2*-knockout and control A549 cells.

a–d Scatter plots comparing the mean PSI values for A549 *CMTR2*-knockout (a, c) and control (b, d) cells treated with 1 µM indisulam or DMSO for 24 h. Significantly associated events (FDR < 0.05) are color-coded in red or blue. SE events (a, b). Retained intron events (c, d).

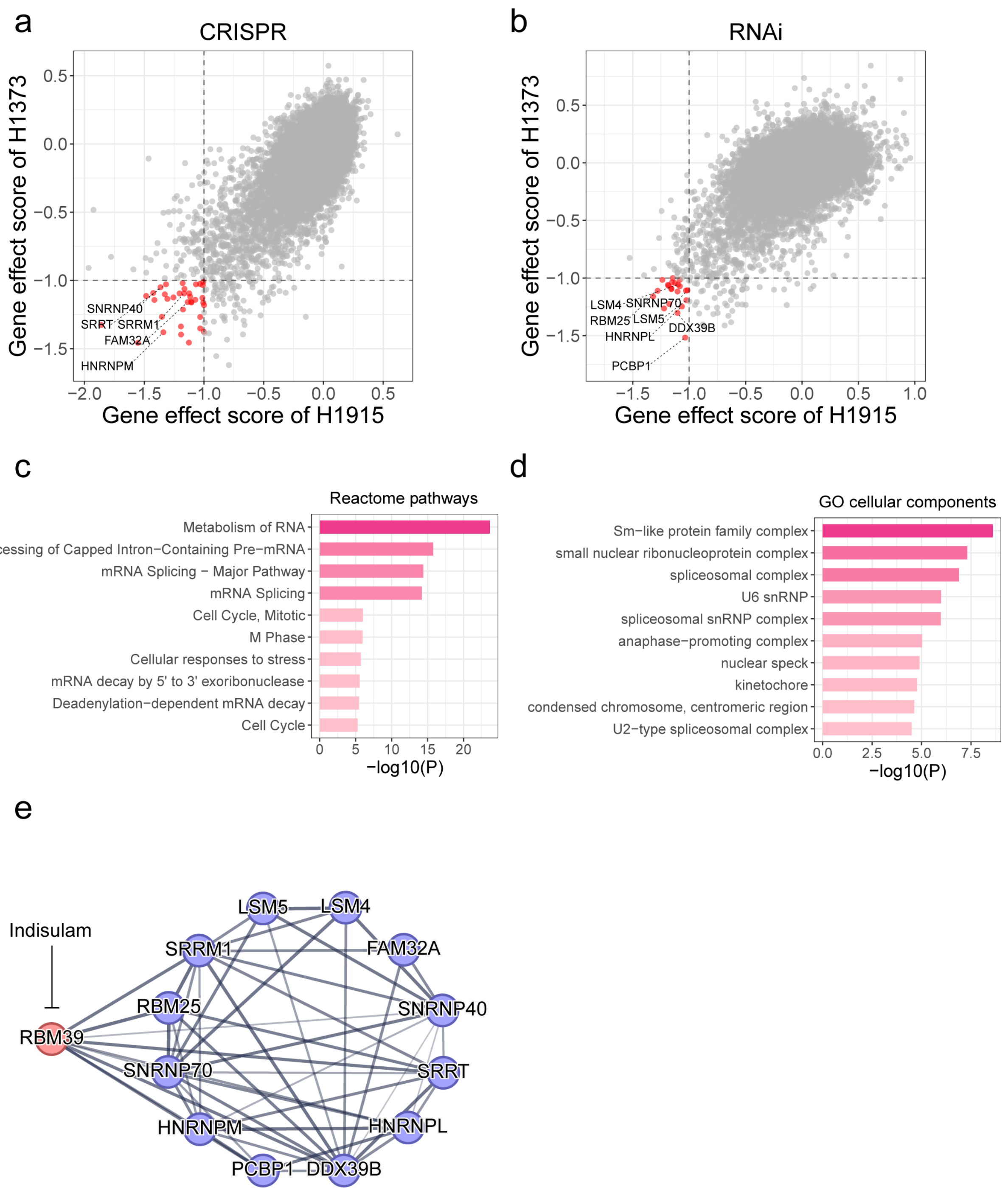

Supplementary Figure 20. *CMTR2*-deficient lung cancer cells are vulnerable to perturbation of splicing-related genes.

a, **b** Scatterplots showing the gene effect (dependency) scores for each gene in NCI-H1373 (y-axis) and NCI-H1915 (x-axis) cells according to CRISPR screening (a) and RNAi screening (b) data from the DepMap portal.

Each point represents a gene included in the datasets. Genes with an average effect score ≤-1.0 in NSCLC cell lines were excluded. **c**, **d** Metascape enrichment analysis of vulnerability genes common to NCI-H1373 and NCI-H1915 cell lines. Bar plots show the top 10 enriched terms in the (c) Reactome pathway and (d) GO cellular component gene sets. **e** STRING network analysis depicting interactions between RBM39, an indisulam target, and genes included in the Reactome 'mRNA Splicing' term identified by CRISPR (a) and RNAi (b) screening.

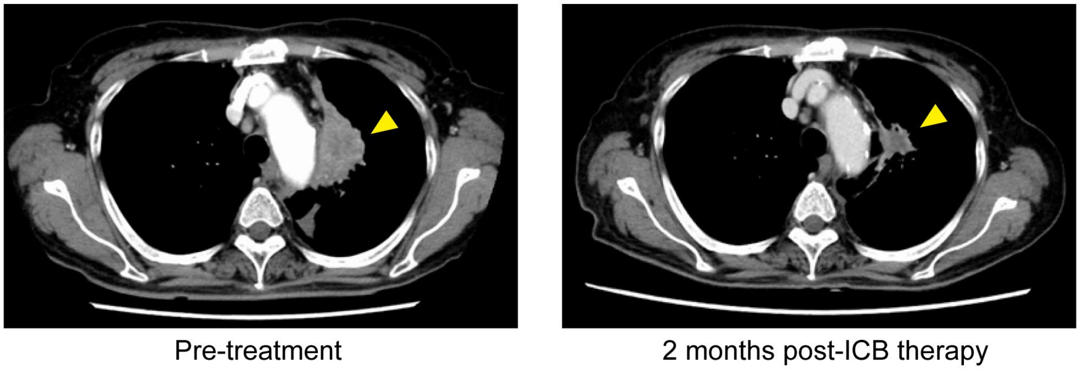

Supplementary Figure 21. Clinical response of a patient with a *CMTR2* truncating mutation to ICB therapy. Chest computed tomography scan images of a LADC patient harboring a *CMTR2* truncating mutation treated with ICB therapy (pembrolizumab) at the NCC. The left panel shows pre-treatment imaging, while the right panel shows imaging at 2 months post-ICB therapy. Yellow arrows indicate the tumor lesion, which demonstrated a partial response to treatment.

CMTR2 cluster	Variant Classification	Protein Change	VAF	OnkoKB Annotation	AM Pathogenicity Score	AM Class	Compound Spliceosomal Mutation
Included	Frame Shift Del	F366Lfs*3	0.2	Unknown	-	-	-
Included	Nonsense Mutation	K32*	0.28	Unknown	-	-	-
Included	Nonsense Mutation	Q113*	0.54	Unknown	-	-	-
Included	Nonsense Mutation	W115*	0.25	Unknown	-	-	-
Included	Nonsense Mutation	G267*	0.18	Unknown	-	-	-
Included	Nonsense Mutation	E558*	0.69	Unknown	-	-	RBM10 P895Rfs*17
Included	Nonsense Mutation	E558*	0.05	Unknown	-	-	-
Included	Splice Site	NULL	0.46	Unknown	-	-	-
Included	Missense Mutation	L291R	0.3	Unknown	0.9667	likely_pathogenic	-
Included	Missense Mutation	L291R	0.15	Unknown	0.9667	likely_pathogenic	-
Included	Missense Mutation	K117N	0.14	Likely Oncogenic	0.9923	likely_pathogenic	-
Included	Missense Mutation	S508P	0.13	Unknown	0.9438	likely_pathogenic	-
Included	Missense Mutation	K275N	0.12	Likely Oncogenic	0.9967	likely_pathogenic	-
Not included	Frame Shift Del	W44Cfs*23	0.24	Unknown	-	-	-
Not included	Missense Mutation	G242V	0.1	Unknown	0.255	likely_benign	-
Not included	Missense Mutation	D235V	0.09	Likely Oncogenic	0.9947	likely_pathogenic	-
Not included	Missense Mutation	F626L	0.12	Unknown	0.6135	likely_pathogenic	-
Not included	Missense Mutation	P147L	0.3	Unknown	0.965	likely_pathogenic	U2AF1 S34F
Not included	Missense Mutation	A684T	0.41	Unknown	0.1136	likely_benign	-
Not included	Missense Mutation	D235V	0.1	Likely Oncogenic	0.9947	likely_pathogenic	-

Supplementary Table 1. Detailed characterization of *CMTR2* mutations in the NCC LADC cohort.

VAF: variant allele frequency; AM: AlphaMissense.

CMTR2 cluster	Variant Classification	Protein Change	VAF	OnkoKB Annotation	AM Pathogenicity Score	AM Class	Compound Spliceosomal Mutation
Included	Frame Shift Del	C511Afs*6	0.18	Unknown	-	-	-
Included	Frame Shift Ins	S423Ifs*2	0.23	Unknown	-	-	-
Included	Nonsense Mutation	G386*	0.42	Unknown	-	-	-
Included	Nonsense Mutation	E391*	0.43	Unknown	-	-	RBM10 E52*
Included	Nonsense Mutation	G528*	0.58	Unknown	-	-	-
Included	Nonsense Mutation	G309*	0.8	Unknown	-	-	RBM10 E187*
Included	Nonsense Mutation	K536*	0.79	Unknown	-	-	-
Included	Nonsense Mutation	E451*	0.19	Unknown	-	-	-
Included	Nonsense Mutation	Q719*	0.51	Unknown	-	-	-
Included	Nonsense Mutation	K732*	0.8	Unknown	-	-	-
Included	Nonsense Mutation	G499*	0.43	Unknown	-	-	-
Included	Splice Site	NULL	0.24	Unknown	-	-	-
Not included	Frame Shift Del	M188Wfs*24	0.45	Unknown	-	-	-
Not included	Nonsense Mutation	E145*	0.65	Unknown	-	-	-
Not included	Frame Shift Del	L497Wfs*9	0.18	Unknown	-	-	-
Not included	Missense Mutation	G484A	0.11	Unknown	0.1292	likely_benign	-
Not included	Missense Mutation	R5I	0.1	Unknown	0.1598	likely_benign	-
Not included	Missense Mutation	H327Y	0.19	Unknown	0.0626	likely_benign	-
Not included	Missense Mutation	E246D	0.2	Unknown	0.4724	ambiguous	-
Not included	Missense Mutation	M727I	0.15	Unknown	0.4088	ambiguous	-
Not included	Missense Mutation	L742V	0.14	Unknown	0.1277	likely_benign	RBM10 Y494*
Not included	Missense Mutation	N155I	0.27	Unknown	0.8984	likely_pathogenic	-
Not included	Missense Mutation	E28K	0.12	Unknown	0.0835	likely_benign	-
Not included	Missense Mutation	C116R	0.22	Unknown	0.9848	likely_pathogenic	-
Not included	Missense Mutation	Q113E	0.3	Unknown	0.3848	ambiguous	-
Not included	Missense Mutation	D457N	0.15	Unknown	0.1021	likely_benign	-
Not included	Nonsense Mutation	C257_E258delins*	0.28	Unknown	-	-	-
Not included	Nonsense Mutation	S717*	0.25	Unknown	-	-	-

Supplementary Table 2. Detailed characterization of *CMTR2* mutations in the TCGA LADC cohort.

Symbol	Cohort	Cancer type	Methods	Mutations	Samples	Q-value combination
CMTR2	TCGA_WXS_LUAD	LADC	oncodrivefml,dndscv,mutpanning	24	21	1.44235E-08
CMTR2	HARTWIG_WGS_NSCLC_2020	NSCLC	oncodrivefml,dndscv	13	12	4.85124E-07

Supplementary Table 3. Driver gene analysis of *CMTR2* across cancer cohorts using the IntOGen web platform.

Analysis results were obtained from the catalog of driver genes and dataset information (2023.05.31). *Q*-value combination represents the significance of the combined output from multiple driver detection methods integrated in the IntOGen pipeline.

	CMTR2 Mutated Cases (N)	CMTR2 Wild-type Cases (N)	Mutation Rate (%)	Odds Ratio	p-value
**Smoker**	42	726	5.5	6.97	4.41E-06
**Never smoker**	4	482	0.8		

Supplementary Table 4. Distribution of *CMTR2* gene mutations among smokers and never-smokers in the TCGA and NCC LADC cohorts.

P-values were obtained using the two-sided Fisher's exact test.

Cell line	Lineage	Lineage Subtype	Oncogenic Driver Alterations	CMTR2 Status	Variant Classification	VAF	Protein Change
NCI-H1373	Lung	Lung Adenocarcinoma	KRAS G12C	Mutated	Frame Shift Del	0.898	p.T339fs
NCI-H1915	Lung	Large Cell Lung Carcinoma	HRAS Q61L	Mutated	Frame Shift Ins	0.997	p.G267fs
MDA-MB-231	Breast	Invasive Breast Carcinoma	KRAS G13D	Mutated	Frame Shift Ins	0.954	p.C427fs
A549	Lung	Lung Adenocarcinoma	KRAS G12S	Wild-type	-	-	-
NCI-H3122	Lung	Lung Adenocarcinoma	EML4-ALK fusion	Wild-type	-	-	-

Supplementary Table 5. *CMTR2* status in cancer cell lines. Information was obtained from the DepMap portal (https://depmap.org/).

	Official Symbol Interactor A	Official Symbol Interactor B	Experimental System	Experimental System Type	Author
BIOGRID	SNRPN	CMTR2	Affinity Capture-MS	physical	Huttlin EL (2017)
BIOGRID	SNRNP70	CMTR2	Affinity Capture-MS	physical	Huttlin EL (2017)
BIOGRID	SNRPE	CMTR2	Affinity Capture-MS	physical	Huttlin EL (2017)
BIOGRID	SNRPF	CMTR2	Affinity Capture-MS	physical	Huttlin EL (2017)
BIOGRID	SNRPG	CMTR2	Affinity Capture-MS	physical	Huttlin EL (2017)
BIOGRID	SNRPB	CMTR2	Affinity Capture-MS	physical	Huttlin EL (2021)
BIOGRID	SNRPG	CMTR2	Affinity Capture-MS	physical	Huttlin EL (2021)
BIOGRID	SNRPD3	CMTR2	Affinity Capture-MS	physical	Huttlin EL (2021)
BIOGRID	SNRPN	CMTR2	Affinity Capture-MS	physical	Huttlin EL (2021)
BIOGRID	SNRPC	CMTR2	Affinity Capture-MS	physical	Huttlin EL (2021)
BIOGRID	SNRPF	CMTR2	Affinity Capture-MS	physical	Huttlin EL (2021)
BIOGRID	SNRPE	CMTR2	Affinity Capture-MS	physical	Huttlin EL (2021)
BIOGRID	SNRNP70	CMTR2	Affinity Capture-MS	physical	Huttlin EL (2021)
BIOGRID	SNRPA	CMTR2	Affinity Capture-MS	physical	Cho NH (2022)
BIOGRID	SNRPB	CMTR2	Affinity Capture-MS	physical	Cho NH (2022)
BIOGRID	SNRPC	CMTR2	Affinity Capture-MS	physical	Cho NH (2022)
BIOGRID	SNRPD2	CMTR2	Affinity Capture-MS	physical	Cho NH (2022)
BIOGRID	SNRPF	CMTR2	Affinity Capture-MS	physical	Cho NH (2022)

Supplementary Table 6. Evidence of protein–protein interactions between CMTR2 and snRNP-associated proteins.

Interaction data were obtained from BioGRID ver. 4.4 (https://thebiogrid.org/). Physical interactions were identified by affinity capture-mass spectrometry as reported by Huttlin et al. 2017[112], Huttlin et al. 2021[113], and Cho et al. 2022[114].

Biological Process (Gene Ontology) Term ID	Term Description	Observed Gene Count	Background Gene Count	FDR	Matching Proteins in Network
GO:0036260	RNA capping	6	21	4.78E-12	SNRPD3,SNRPF,CMTR2,SNRPG,SNRPE,SNRPB
GO:0006397	mRNA processing	9	455	1.61E-11	SNRPD3,SNRPC,SNRPF,CMTR2,SNRPG,SNRPE, SNRPB,SNRNP70,SNRPN
GO:0000387	Spliceosomal snRNP assembly	6	39	3.07E-11	SNRPD3,SNRPC,SNRPF,SNRPG,SNRPE,SNRPB
GO:0000398	mRNA splicing, via spliceosome	8	245	3.07E-11	SNRPD3,SNRPC,SNRPF,SNRPG,SNRPE,SNRPB, SNRNP70,SNRPN
GO:0036261	7-methylguanosine cap hypermethylation	5	8	3.07E-11	SNRPD3,SNRPF,SNRPG,SNRPE,SNRPB
GO:0000245	Spliceosomal complex assembly	6	76	5.62E-10	SNRPD3,SNRPC,SNRPF,SNRPG,SNRPE,SNRPB
GO:1903241	U2-type prespliceosome assembly	5	24	8.58E-10	SNRPD3,SNRPF,SNRPG,SNRPE,SNRPB
GO:0001510	RNA methylation	6	89	1.07E-09	SNRPD3,SNRPF,CMTR2,SNRPG,SNRPE,SNRPB

Supplementary Table 7. Functional enrichment analysis of CMTR2 protein interactions.

Analysis results were obtained from STRING ver. 12.0 (https://string-db.org/).

Sample_ID	Sex	Smoking Pack Years	Pathology	Sample Origin	Oncogenic Driver Alterations	CMTR2 mutation	Ref allele	Alt allele	VAF	Protein Change
KOR165	Male	0	LADC	Resected tumor	EGFR L858R	Not detected	-	-	-	-
KOR391	Female	0	LADC	Ascites	EGFR ELREA746del	Not detected	-	-	-	-
KOR558	Male	62.5	LADC	Pleural effusion	EGFR G719A	Nonsense	C	A	0.968	p.E43*

Supplementary Table 8. Characteristics and mutation profiles of lung cancer organoids.
