# Supplementary material for: Mutation of CMTR2 in Lung Adenocarcinoma Alters RNA Alternative Splicing and Reveals Therapeutic Vulnerabilities
Source: Nat Commun. 2025 Nov 6;16:9754. doi: 10.1038/s41467-025-64821-0 (PMC12592727; doi:10.1038/s41467-025-64821-0)
Supplement: Supplementary file 2 — Description of Additional Supplementary Files [file 41467_2025_64821_MOESM2_ESM.pdf]

Description of Additional Supplementary Files

Supplementary Data 1: Detailed analysis of common skipped exon events across datasets from human samples and cancer cell lines.

Supplementary Data 2: Identification of CMTR2-interacting proteins by FLAG immunoprecipitation and NanoLC-MS/MS.

Supplementary Data 3: Clinical characteristics and mutation profile of a case with a CMTR2 truncating mutation treated with ICB Therapy at NCC.
