# Supplementary material for: Mutation of CMTR2 in Lung Adenocarcinoma Alters RNA Alternative Splicing and Reveals Therapeutic Vulnerabilities
Source: Nat Commun. 2025 Nov 6;16:9754. doi: 10.1038/s41467-025-64821-0 (PMC12592727; doi:10.1038/s41467-025-64821-0)
Supplement: Supplementary file 6 — Reporting Summary [file 41467_2025_64821_MOESM6_ESM.pdf]

nature portfolio

Corresponding author(s): Takashi Kohno, Takashi Nakaoku

Last updated by author(s): Sep 20, 2025

Reporting Summary

Nature Portfolio wishes to improve the reproducibility of the work that we publish. This form provides structure for consistency and transparency in reporting. For further information on Nature Portfolio policies, see our Editorial Policies and the Editorial Policy Checklist.

Statistics

For all statistical analyses, confirm that the following items are present in the figure legend, table legend, main text, or Methods section.

n/a	Confirmed	
[ ]	[x]	The exact sample size (*n*) for each experimental group/condition, given as a discrete number and unit of measurement
[ ]	[x]	A statement on whether measurements were taken from distinct samples or whether the same sample was measured repeatedly
[ ]	[x]	The statistical test(s) used AND whether they are one- or two-sided<br>*Only common tests should be described solely by name; describe more complex techniques in the Methods section.*
[ ]	[x]	A description of all covariates tested
[ ]	[x]	A description of any assumptions or corrections, such as tests of normality and adjustment for multiple comparisons
[ ]	[x]	A full description of the statistical parameters including central tendency (e.g. means) or other basic estimates (e.g. regression coefficient) AND variation (e.g. standard deviation) or associated estimates of uncertainty (e.g. confidence intervals)
[ ]	[x]	For null hypothesis testing, the test statistic (e.g. *F*, *t*, *r*) with confidence intervals, effect sizes, degrees of freedom and *P* value noted<br>*Give P values as exact values whenever suitable.*
[x]	[ ]	For Bayesian analysis, information on the choice of priors and Markov chain Monte Carlo settings
[x]	[ ]	For hierarchical and complex designs, identification of the appropriate level for tests and full reporting of outcomes
[x]	[ ]	Estimates of effect sizes (e.g. Cohen's *d*, Pearson's *r*), indicating how they were calculated

Our web collection on statistics for biologists contains articles on many of the points above.

Software and code

Policy information about availability of computer code

Data collection	EVOS M7000 Imaging System and Celleste 6 Image Analysis Software (Thermo Fisher Scientific) were used for the colony formation assay, LAS3000 and FUSION FX imaging systems (Quansys Biosciences and Vilber Lourmat, respectively) were used for western blotting, an Agilent TapeStation 4150 system (Agilent Technologies) was used for RT-PCR, and BZ-X800 (Keyence) was used for imaging lung cancer organoids.
Data analysis	R ver.4.2.2<br>GraphPad Prism ver.9.5.1<br>STAR ver.2.7.9a<br>STAR-Fusion ver.1.11.0<br>StringTie ver.2.0.4<br>RSEM ver.1.3.3<br>basecaller ver. 0.9.1+c8c2c9f<br>chopper<br>minimap2 ver. 2.28-r1209<br>bambu ver.3.8.3<br>rMATS turbo ver.4.1.2<br>Rtsne ver.0.16<br>DESeq2 ver.1.36.0<br>ggseqlogo ver.0.2<br>GSVA ver.3.2.0<br>Parabricks ver.3.1.3

BWA ver.0.7.15
GATK ver.4.1.0 and ver.4.1.2.0
TCGAmutations ver.0.3.0
MutPanning ver.2.0
OncodriveFML ver.2.1.3
DriverPower ver.1.0.2
OncodriveCLUSTL ver.1.1.3
PyClone-VI ver.0.1.1
Facets ver.0.6.2
ClonEvol ver.0.99.11
ComplexHeatmap ver.2.12.1

For manuscripts utilizing custom algorithms or software that are central to the research but not yet described in published literature, software must be made available to editors and reviewers. We strongly encourage code deposition in a community repository (e.g. GitHub). See the Nature Portfolio guidelines for submitting code & software for further information.

Data

Policy information about availability of data

All manuscripts must include a data availability statement. This statement should provide the following information, where applicable:

- Accession codes, unique identifiers, or web links for publicly available datasets
- A description of any restrictions on data availability
- For clinical datasets or third party data, please ensure that the statement adheres to our policy

The RNA-seq data and the whole-exome sequencing data generated in this study have been deposited in the Japanese Genotype–phenotype Archive (JGA) under accession codes JGAS000756 [https://ddbj.nig.ac.jp/search/entry/jga-study/JGAS000756] and JGAD000897 [https://ddbj.nig.ac.jp/search/entry/jga-dataset/JGAD000897]. Access to the RNA-seq data and the whole-exome sequencing data from the NCC cohort are restricted to protect patient privacy in accordance with the Japanese data privacy laws. Access can be obtained by submitting a research proposal to the National Bioscience Database Center (NBDC) using accession code hum0445 [https://humandbs.dbcls.jp/en/hum0445-v1]. Data users shall apply for data use in accordance with the data use application procedures (https://humandbs.biosciencedbc.jp/en/data-use). Data must be used in compliance with NBDC Guidelines for Human Data Sharing, and NBDC Security Guidelines for Human Data (for Data Users), which are available at https://humandbs.dbcls.jp/en/guidelines/data-sharing-guidelines and https://humandbs.dbcls.jp/en/guidelines/security-guidelines-for-users. A summary of how to use controlled access data is available at https://humandbs.dbcls.jp/en/data-use. To request access, contact https://humandbs.ddbj.nig.ac.jp/nbdc/application/. Restrictions on data access and usage are described in the NBDC data sharing guidelines, available at https://humandbs.dbcls.jp/en/guidelines/data-sharing-guidelines. The expected time frame for responses to access requests is within a couple of weeks, and data will be available for the duration of the approved research period. The RNA-seq data from the cell line experiments generated in this study have been deposited in the DDBJ Sequence Read Archive (DRA) under accession number PRJDB18501 [https://ddbj.nig.ac.jp/search/entry/bioproject/PRJDB18501]. The mass spectrometry proteomics data generated in this study have been deposited in the ProteomeXchange Consortium via the jPOST partner repository under accession code PXD067711 [http://proteomecentral.proteomexchange.org/cgi/GetDataset?ID=PXD067711]. The protein structural data used in this study are available in the PDB database under accession code 7B0Y [https://www.rcsb.org/structure/7B0Y], and in the AlphaFold database under accession code Q8IYT2 [https://alphafold.ebi.ac.uk/entry/Q8IYT2]. The chemical structure data used in this study are available in the ChEBI database under accession code CHEBI:167614 [https://www.ebi.ac.uk/chebi/beta/CHEBI:167614]. Source data are provided with this paper.

Research involving human participants, their data, or biological material

Policy information about studies with human participants or human data. See also policy information about sex, gender (identity/presentation), and sexual orientation and race, ethnicity and racism.

Reporting on sex and gender	Sex was self-reported during patient enrollment, and confirmed by reference to medical records. Gender identity was not assessed specifically in this study. No sex or gender analysis was carried out because the research questions focused on the molecular mechanisms of lung cancer, which are not thought to differ according to sex or gender.
Reporting on race, ethnicity, or other socially relevant groupings	The human samples used for RNA splicing analysis in this study were obtained from Japanese lung cancer patients treated at the National Cancer Center Hospital in Tokyo, Japan.
Population characteristics	The NCC cohort of 1017 cases was diagnosed with lung cancer at the NCC Hospital in Tokyo, Japan, between 2011 and 2017. The cohort comprised 568 males and 449 females. The median age was 66 years (range: 26-89 years; males: median 65 years, range 26-89; females: median 66 years, range 35-88). Written informed consent to participate and to publish potentially identifiable information (including combinations of indirect identifiers) was obtained from all participants in accordance with institutional policies.
Recruitment	Participants were selected from patients diagnosed with lung cancer at the NCC Hospital (Japan) between 2011 and 2017, based on provision of informed consent and availability of sequenceable tissue samples from surgical resection. This sample selection approach may introduce biases, including geographic limitations, restriction to surgical candidates with adequate tissue quality, and potential over-representation of patients with earlier-stage disease. These factors may limit generalizability of the findings to broader lung cancer populations.
Ethics oversight	The study using patient samples from the NCC cohort was approved by the Institutional Review Board of the NCC (IORG0002238) (2005-109).

Note that full information on the approval of the study protocol must also be provided in the manuscript.

Field-specific reporting

Please select the one below that is the best fit for your research. If you are not sure, read the appropriate sections before making your selection.

- [x] Life sciences
- [ ] Behavioural & social sciences
- [ ] Ecological, evolutionary & environmental sciences

For a reference copy of the document with all sections, see nature.com/documents/nr-reporting-summary-flat.pdf

Life sciences study design

All studies must disclose on these points even when the disclosure is negative.

Sample size	Selection of sample sizes was based on prior experience of similar studies, practical considerations (including cost and specimen availability), and availability of high quality sequencing data. While formal statistical power calculations were not performed, the chosen sample sizes are consistent with those used in comparable genomic studies and are sufficient to detect biologically meaningful effects.
Data exclusions	No samples were excluded from the analysis of RNA splicing conducted using RNA sequencing data from human lung cancer specimens. To maintain data consistency, only poly-A captured RNA sequencing datasets were analyzed.
Replication	Most experimental results described in the paper are based on independent biological replicates, and were reproduced successfully; however, some experiments, including mass spectrometry, could not be replicated due to cost limitations. For these experiments, representative images obtained from multiple experiments with similar results are presented. Further details regarding replication and sample sizes are provided in the individual figure legends.
Randomization	For the mouse experiments, animals were assigned assigned to experimental and control groups before inhibitor administration. For the other experiments, randomization was not performed as they involved controlled comparisons between genetically defined cell lines under standardized laboratory conditions.
Blinding	RNA splicing analyses were performed by investigators blinded to specimen background information. Investigators were not blinded to the treatment groups or genotypes used in the experimental studies (in vivo and in vitro) reported in this manuscript, as in these cases blinding was considered not to affect the measurement of results.

Behavioural & social sciences study design

All studies must disclose on these points even when the disclosure is negative.

Study description	*Briefly describe the study type including whether data are quantitative, qualitative, or mixed-methods (e.g. qualitative cross-sectional, quantitative experimental, mixed-methods case study).*
Research sample	*State the research sample (e.g. Harvard university undergraduates, villagers in rural India) and provide relevant demographic information (e.g. age, sex) and indicate whether the sample is representative. Provide a rationale for the study sample chosen. For studies involving existing datasets, please describe the dataset and source.*
Sampling strategy	*Describe the sampling procedure (e.g. random, snowball, stratified, convenience). Describe the statistical methods that were used to predetermine sample size OR if no sample-size calculation was performed, describe how sample sizes were chosen and provide a rationale for why these sample sizes are sufficient. For qualitative data, please indicate whether data saturation was considered, and what criteria were used to decide that no further sampling was needed.*
Data collection	*Provide details about the data collection procedure, including the instruments or devices used to record the data (e.g. pen and paper, computer, eye tracker, video or audio equipment) whether anyone was present besides the participant(s) and the researcher, and whether the researcher was blind to experimental condition and/or the study hypothesis during data collection.*
Timing	*Indicate the start and stop dates of data collection. If there is a gap between collection periods, state the dates for each sample cohort.*
Data exclusions	*If no data were excluded from the analyses, state so OR if data were excluded, provide the exact number of exclusions and the rationale behind them, indicating whether exclusion criteria were pre-established.*
Non-participation	*State how many participants dropped out/declined participation and the reason(s) given OR provide response rate OR state that no participants dropped out/declined participation.*
Randomization	*If participants were not allocated into experimental groups, state so OR describe how participants were allocated to groups, and if allocation was not random, describe how covariates were controlled.*

Ecological, evolutionary & environmental sciences study design

All studies must disclose on these points even when the disclosure is negative.

Study description	*Briefly describe the study. For quantitative data include treatment factors and interactions, design structure (e.g. factorial, nested,*

hierarchical), nature and number of experimental units and replicates.

Research sample	*Describe the research sample (e.g. a group of tagged Passer domesticus, all Stenocereus thurberi within Organ Pipe Cactus National Monument), and provide a rationale for the sample choice. When relevant, describe the organism taxa, source, sex, age range and any manipulations. State what population the sample is meant to represent when applicable. For studies involving existing datasets, describe the data and its source.*
Sampling strategy	*Note the sampling procedure. Describe the statistical methods that were used to predetermine sample size OR if no sample-size calculation was performed, describe how sample sizes were chosen and provide a rationale for why these sample sizes are sufficient.*
Data collection	*Describe the data collection procedure, including who recorded the data and how.*
Timing and spatial scale	*Indicate the start and stop dates of data collection, noting the frequency and periodicity of sampling and providing a rationale for these choices. If there is a gap between collection periods, state the dates for each sample cohort. Specify the spatial scale from which the data are taken*
Data exclusions	*If no data were excluded from the analyses, state so OR if data were excluded, describe the exclusions and the rationale behind them, indicating whether exclusion criteria were pre-established.*
Reproducibility	*Describe the measures taken to verify the reproducibility of experimental findings. For each experiment, note whether any attempts to repeat the experiment failed OR state that all attempts to repeat the experiment were successful.*
Randomization	*Describe how samples/organisms/participants were allocated into groups. If allocation was not random, describe how covariates were controlled. If this is not relevant to your study, explain why.*
Blinding	*Describe the extent of blinding used during data acquisition and analysis. If blinding was not possible, describe why OR explain why blinding was not relevant to your study.*

Did the study involve field work? ☐ Yes ☒ No

Field work, collection and transport

Field conditions	*Describe the study conditions for field work, providing relevant parameters (e.g. temperature, rainfall).*
Location	*State the location of the sampling or experiment, providing relevant parameters (e.g. latitude and longitude, elevation, water depth).*
Access & import/export	*Describe the efforts you have made to access habitats and to collect and import/export your samples in a responsible manner and in compliance with local, national and international laws, noting any permits that were obtained (give the name of the issuing authority, the date of issue, and any identifying information).*
Disturbance	*Describe any disturbance caused by the study and how it was minimized.*

Reporting for specific materials, systems and methods

We require information from authors about some types of materials, experimental systems and methods used in many studies. Here, indicate whether each material, system or method listed is relevant to your study. If you are not sure if a list item applies to your research, read the appropriate section before selecting a response.

Materials & experimental systems

n/a	Involved in the study	
☐	☒	Antibodies
☐	☒	Eukaryotic cell lines
☒	☐	Palaeontology and archaeology
☐	☒	Animals and other organisms
☒	☐	Clinical data
☒	☐	Dual use research of concern
☒	☐	Plants

Methods

n/a	Involved in the study	
☒	☐	ChIP-seq
☒	☐	Flow cytometry
☒	☐	MRI-based neuroimaging

Antibodies

Antibodies used	Anti-mouse PD-1 (Ultra-LEAF Purified anti-mouse CD279 (PD-1); catalog No. 114122; clone RMP1-14; lot B434686) and isotype control (Ultra-LEAF Purified Rat IgG2a, κ Isotype Ctrl; catalog No. 400574; clone RTK2758; lot B449464) antibodies were purchased from BioLegend and used for in vivo treatments. The following primary antibodies were used for immunoblot analysis: anti-β-actin (1:1000; catalog No. 3700, clone 8H10D10; lot 13, Cell Signaling Technology); anti-cleaved PARP (1:1000; catalog No. 5625, clone D64E10; lot 13, Cell Signaling Technology); anti-RBM39 (1:1000; catalog No. HPA001591, polyclonal; lot 000044370, Sigma-Aldrich);

	anti-FLAG (1:3000; catalog No. F1804, clone M2; lot 0000278731, Sigma-Aldrich); anti-CMTR2 (1:500; catalog No. PA5-61696, polyclonal; lot YG3983628A, Thermo Fisher Scientific); and anti-SNRNP70 (1:1000; catalog No. SC390899, clone C-3; lot G2122, Santa Cruz Biotechnology).
Validation	All primary antibodies used in this study are available commercially and have been verified by the respective manufacturers. Detailed validation information and pertinent citations can be accessed through official websites: Cell Signaling Technology (https://www.cellsignal.com/), Sigma-Aldrich (https://www.sigmaaldrich.com/), Thermo Fisher Scientific (https://www.thermofisher.com/), and Santa Cruz Biotechnology (https://www.scbt.com/).

Eukaryotic cell lines

Policy information about cell lines and Sex and Gender in Research

Cell line source(s)	A549 (CCL-185), NCI-H1915 (CRL-5904), NCI-H1373 (CRL-5866), and LLC (CRL-1642) cells were purchased from the American Type Culture Collection. NCI-H3122 was provided by Dr William Pao (Vanderbilt University, Nashville, TN, USA). Flp-in T-REx 293 cells (R78007) were purchased from Thermo Fisher Scientific. A549 (male), NCI-H3122 (male), NCI-H1915 (female), NCI-H1373 (male), and Flp-in T-REx 293 (female) are human-derived cell lines. LLC cells are mouse-derived.
Authentication	All cell lines were authenticated using Short Tandem Repeat (STR) profiling (Promega).
Mycoplasma contamination	All cell lines tested negative for mycoplasma using a MycoAlert detection kit (Lonza).
Commonly misidentified lines (See ICLAC register)	No commonly misidentified cell lines were used in this study.

Palaeontology and Archaeology

Specimen provenance	*Provide provenance information for specimens and describe permits that were obtained for the work (including the name of the issuing authority, the date of issue, and any identifying information). Permits should encompass collection and, where applicable, export.*
Specimen deposition	*Indicate where the specimens have been deposited to permit free access by other researchers.*
Dating methods	*If new dates are provided, describe how they were obtained (e.g. collection, storage, sample pretreatment and measurement), where they were obtained (i.e. lab name), the calibration program and the protocol for quality assurance OR state that no new dates are provided.*

☐ Tick this box to confirm that the raw and calibrated dates are available in the paper or in Supplementary Information.

Ethics oversight	*Identify the organization(s) that approved or provided guidance on the study protocol, OR state that no ethical approval or guidance was required and explain why not.*

Note that full information on the approval of the study protocol must also be provided in the manuscript.

Animals and other research organisms

Policy information about studies involving animals; ARRIVE guidelines recommended for reporting animal research, and Sex and Gender in Research

Laboratory animals	Female BALB/c-nu mice (Charles River, RRID: IMSR_CRL:194; n=32 in total) and C57BL/6J mice (The Jackson Laboratory, RRID: IMSR_JAX:000664; n=80 in total), all aged 5 weeks, were used for the xenograft and syngeneic models, respectively. Mice were maintained under standard, strictly controlled specific-pathogen-free conditions: temperature 22 ± 0.5°C, relative humidity 55 ± 10%, and a 12-h light/dark cycle (lights on 08:00-20:00).
Wild animals	This study did not involve wild animals.
Reporting on sex	Female mice were used exclusively to minimize aggressive and dominance-related behaviors that could introduce experimental variability.
Field-collected samples	This study did not involve samples collected from the field.
Ethics oversight	All animal experiments were approved by the Committee for Ethics of Animal Experimentation of the NCC (approval numbers: A277bM2-23 and A277bM3-25) .

Note that full information on the approval of the study protocol must also be provided in the manuscript.

Clinical data

Policy information about clinical studies

All manuscripts should comply with the ICMJE guidelines for publication of clinical research and a completed CONSORT checklist must be included with all submissions.

Clinical trial registration	*Provide the trial registration number from ClinicalTrials.gov or an equivalent agency.*
Study protocol	*Note where the full trial protocol can be accessed OR if not available, explain why.*
Data collection	*Describe the settings and locales of data collection, noting the time periods of recruitment and data collection.*
Outcomes	*Describe how you pre-defined primary and secondary outcome measures and how you assessed these measures.*

Dual use research of concern

Policy information about dual use research of concern

Hazards

Could the accidental, deliberate or reckless misuse of agents or technologies generated in the work, or the application of information presented in the manuscript, pose a threat to:

No	Yes	
☐	☐	Public health
☐	☐	National security
☐	☐	Crops and/or livestock
☐	☐	Ecosystems
☐	☐	Any other significant area

Experiments of concern

Does the work involve any of these experiments of concern:

No	Yes	
☐	☐	Demonstrate how to render a vaccine ineffective
☐	☐	Confer resistance to therapeutically useful antibiotics or antiviral agents
☐	☐	Enhance the virulence of a pathogen or render a nonpathogen virulent
☐	☐	Increase transmissibility of a pathogen
☐	☐	Alter the host range of a pathogen
☐	☐	Enable evasion of diagnostic/detection modalities
☐	☐	Enable the weaponization of a biological agent or toxin
☐	☐	Any other potentially harmful combination of experiments and agents

Plants

Seed stocks	Not applicable
Novel plant genotypes	Not applicable
Authentication	Not applicable

ChIP-seq

Data deposition

☐ Confirm that both raw and final processed data have been deposited in a public database such as GEO.

☐ Confirm that you have deposited or provided access to graph files (e.g. BED files) for the called peaks.

Data access links<br>*May remain private before publication.*	*For "Initial submission" or "Revised version" documents, provide reviewer access links. For your "Final submission" document, provide a link to the deposited data.*
Files in database submission	*Provide a list of all files available in the database submission.*
Genome browser session<br>(e.g. UCSC)	*Provide a link to an anonymized genome browser session for "Initial submission" and "Revised version" documents only, to enable peer review. Write "no longer applicable" for "Final submission" documents.*

Methodology

Replicates	*Describe the experimental replicates, specifying number, type and replicate agreement.*
Sequencing depth	*Describe the sequencing depth for each experiment, providing the total number of reads, uniquely mapped reads, length of reads and whether they were paired- or single-end.*
Antibodies	*Describe the antibodies used for the ChIP-seq experiments; as applicable, provide supplier name, catalog number, clone name, and lot number.*
Peak calling parameters	*Specify the command line program and parameters used for read mapping and peak calling, including the ChIP, control and index files used.*
Data quality	*Describe the methods used to ensure data quality in full detail, including how many peaks are at FDR 5% and above 5-fold enrichment.*
Software	*Describe the software used to collect and analyze the ChIP-seq data. For custom code that has been deposited into a community repository, provide accession details.*

Flow Cytometry

Plots

Confirm that:

☐ The axis labels state the marker and fluorochrome used (e.g. CD4-FITC).

☐ The axis scales are clearly visible. Include numbers along axes only for bottom left plot of group (a 'group' is an analysis of identical markers).

☐ All plots are contour plots with outliers or pseudocolor plots.

☐ A numerical value for number of cells or percentage (with statistics) is provided.

Methodology

Sample preparation	*Describe the sample preparation, detailing the biological source of the cells and any tissue processing steps used.*
Instrument	*Identify the instrument used for data collection, specifying make and model number.*
Software	*Describe the software used to collect and analyze the flow cytometry data. For custom code that has been deposited into a community repository, provide accession details.*
Cell population abundance	*Describe the abundance of the relevant cell populations within post-sort fractions, providing details on the purity of the samples and how it was determined.*
Gating strategy	*Describe the gating strategy used for all relevant experiments, specifying the preliminary FSC/SSC gates of the starting cell population, indicating where boundaries between "positive" and "negative" staining cell populations are defined.*

☐ Tick this box to confirm that a figure exemplifying the gating strategy is provided in the Supplementary Information.

Magnetic resonance imaging

Experimental design

Design type	*Indicate task or resting state; event-related or block design.*
Design specifications	*Specify the number of blocks, trials or experimental units per session and/or subject, and specify the length of each trial or block (if trials are blocked) and interval between trials.*

Behavioral performance measures

State number and/or type of variables recorded (e.g. correct button press, response time) and what statistics were used to establish that the subjects were performing the task as expected (e.g. mean, range, and/or standard deviation across subjects).

Acquisition

Imaging type(s)

Specify: functional, structural, diffusion, perfusion.

Field strength

Specify in Tesla

Sequence & imaging parameters

Specify the pulse sequence type (gradient echo, spin echo, etc.), imaging type (EPI, spiral, etc.), field of view, matrix size, slice thickness, orientation and TE/TR/flip angle.

Area of acquisition

State whether a whole brain scan was used OR define the area of acquisition, describing how the region was determined.

Diffusion MRI ☐ Used ☐ Not used

Preprocessing

Preprocessing software

Provide detail on software version and revision number and on specific parameters (model/functions, brain extraction, segmentation, smoothing kernel size, etc.).

Normalization

If data were normalized/standardized, describe the approach(es): specify linear or non-linear and define image types used for transformation OR indicate that data were not normalized and explain rationale for lack of normalization.

Normalization template

Describe the template used for normalization/transformation, specifying subject space or group standardized space (e.g. original Talairach, MNI305, ICBM152) OR indicate that the data were not normalized.

Noise and artifact removal

Describe your procedure(s) for artifact and structured noise removal, specifying motion parameters, tissue signals and physiological signals (heart rate, respiration).

Volume censoring

Define your software and/or method and criteria for volume censoring, and state the extent of such censoring.

Statistical modeling & inference

Model type and settings

Specify type (mass univariate, multivariate, RSA, predictive, etc.) and describe essential details of the model at the first and second levels (e.g. fixed, random or mixed effects; drift or auto-correlation).

Effect(s) tested

Define precise effect in terms of the task or stimulus conditions instead of psychological concepts and indicate whether ANOVA or factorial designs were used.

Specify type of analysis: ☐ Whole brain ☐ ROI-based ☐ Both

Statistic type for inference

(See Eklund et al. 2016)

Specify voxel-wise or cluster-wise and report all relevant parameters for cluster-wise methods.

Correction

Describe the type of correction and how it is obtained for multiple comparisons (e.g. FWE, FDR, permutation or Monte Carlo).

Models & analysis

n/a	Involved in the study
☐	☐ Functional and/or effective connectivity
☐	☐ Graph analysis
☐	☐ Multivariate modeling or predictive analysis

Functional and/or effective connectivity

Report the measures of dependence used and the model details (e.g. Pearson correlation, partial correlation, mutual information).

Graph analysis

Report the dependent variable and connectivity measure, specifying weighted graph or binarized graph, subject- or group-level, and the global and/or node summaries used (e.g. clustering coefficient, efficiency, etc.).

Multivariate modeling and predictive analysis

Specify independent variables, features extraction and dimension reduction, model, training and evaluation metrics.
